# Anti-inflammatory effects of mesenchymal stem cell-conditioned media inhibited macrophages activation in vitro

**DOI:** 10.1038/s41598-022-08398-4

**Published:** 2022-03-19

**Authors:** Quan-He Jin, Hyung-Keun Kim, Ju-Yong Na, Cheng Jin, Jong-Keun Seon

**Affiliations:** 1 Department of Orthopaedic Surgery, Chonnam National University Medical School and Hospital, 322 Seoyang-ro, Hwasun-eup, Hwasun-gun, Jeollanam-do Republic of Korea; 2 Department of Orthopaedic Surgery, Affiliated Sir Run Run Hospital of Nanjing Medical University, Nanjing, Jiangsu People’s Republic of China; 3 Korea Biomedical Materials and Devices Innovation Research Center, Chonnam National University Hospital, Gwangju, Republic of Korea; 4 Department of Orthopaedic Surgery, Zhoushan Hospital, Zhoushan, Zhejiang People’s Republic of China

**Keywords:** Cell biology, Stem cells

## Abstract

The immunomodulatory effects of mesenchymal stem cells (MSCs) on macrophages have been reported, however, the underlying mechanism remains unknown. Therefore, this study aimed to investigate the anti-inflammatory effects of MSCs on lipopolysaccharide (LPS)-stimulated macrophages and the subsequent downregulation of their inflammatory mediators. Macrophages were treated with conditioned media from MSCs, without a subsequent change of MSCs responding to the inflammation state. This study also evaluated whether the interleukin (IL) 4 stimulation of MSCs can improve their anti-inflammatory effects. Results demonstrated that the MSC-conditioned medium (MSC-CM) stimulated with IL4 significantly inhibited inducible nitric oxide synthase (iNOS) and cyclooxygenase-2 (COX-2) protein expression of LPS-activated macrophages. MSC-CM treatment inhibited the mRNA transcription of the cytokines IL1β and IL6, the chemokines C–C motif ligand (CCL) 2, CCL3, CCL4, and CCL5, and the chemokine receptors CCR2 and CCR5, in LPS-stimulated macrophages. As revealed through western blot and immunofluorescence analyses, the phosphorylation of p38, JNK, and ERK MAPKs, as well as phosphorylation of NF-κB in stimulated macrophages, were also inhibited by the MSC-CM. Further, more potent anti-inflammatory effects were observed with the IL4-stimulated cells, compared with those observed with the non-stimulated cells. The MSC-CM demonstrated a potent anti-inflammatory effect on LPS-activated macrophages, while the IL4 stimulation improved this effect. These findings indicate that MSCs could exert anti-inflammatory effects on macrophages, and may be considered as a therapeutic agent in inflammation treatment.

## Introduction

Inflammation is a protective response mediated by immune cells and molecular mediators toward invasive pathogens that cause infection and tissue damage. However, inflammation is also related to many pathophysiological processes^1^. Some diseases, such as osteoarthritis (OA)^2^, rheumatoid arthritis^3^, inflammatory bowel disease^4^, diabetes^5^, and acute lung injury^6^ have been reported to be related to inflammation induced by immune cells and molecular mediators.

Immune cells, such as macrophages, produce inducible nitric oxide synthase (iNOS) and cyclooxygenase-2 (COX-2), to induce inflammation^7^. Activated macrophages can secrete the cytokines interleukin (IL) 6, IL1β, and TNF-α, which enhance inflammation^8,9^. Chemokines, such as the C–C motif ligand (CCL) 2, CCL3, and CCL5, are also produced to regulate the chemotactic movement of immune cells^10–12^. These inflammatory signals often rely on the activation of Toll-like receptors^13,14^, and perform a major role in inflammatory disease.

The use of stem cells might offer the most promising cell therapy strategy for the biological treatment of inflammation^15–17^. Mesenchymal stem cells (MSCs) currently represent an important immunotherapeutic cell group, and there are several possible applications of MSCs in the treatment of immune function-related diseases^18–20^. The regulatory network of the factors inducing the generation of the regulatory immune cells is a characteristic of MSCs participating in immune homeostasis, making them conducive for immunomodulation^21–23^. MSCs are circumstance-sensitive cells, as they respond to extracellular stimuli by inducing various interventions that are specifically sensitive to alterations in signals^24,25^. Upon exposure to pro-inflammatory factors, MSC-mediated immunomodulation is upgraded^17,26^.

MSC-conditioned media (MSC-CM) refer to the supernatants of an MSC culture, containing various cytokines and extracellular vesicles secreted by MSCs which can regulate the immune response^27,28^. Takafuji et al. revealed that a conditioned medium from cultured MSCs downregulated the expression of tumor necrosis factor (TNF)-α and interleukin (IL) 6 in macrophages by suppressing the mitogen-activated protein kinase (MAPK) and nuclear factor kappa-B (NF-κB) pathways, while also decreasing the expression of M2 markers^27^. MSC-CM was also reported to reduce the expression of MIP-2, a form of chemoattractant, and IL-6 in the tissues of an acute lung injury model^6^. MSC-CM-isolated extracellular vesicles were also reported to downregulate IL6 and CCL2 expression in a skeletal muscle injury model^29^. In addition, Previous studies showed that, as the circumstance sensitive cells, upon exposure to pro-inflammatory factors, MSC-mediated immunomodulation was regulated^17,26^. Therefore, due to the immunomodulatory effects of MSCs, we wondered whether IL4 treatment of MSCs would have less or more anti-inflammatory effects on activated macrophages.

In this study, we hypothesized that several paracrine cytokines exist in stem cell culture media that may facilitate the immunomodulation of macrophages. The IL4 treatment of stem cells could lead to a greater production of those compounds which improve the anti-inflammatory effects of the stem cells on macrophages stimulated by lipopolysaccharide (LPS). In this study, the anti-inflammatory effects of MSC-CM on LPS-stimulated macrophages were investigated, and the signal transduction pathways involved in the immunomodulation of MSC-CM were analyzed.

## Results

### MSC-CM decreased the production of COX-2 and iNOS

The MSC-CM did not exert any toxic effects on macrophages (Supplementary Fig. 1). The protein expression of COX-2 and iNOS in the LPS-stimulated macrophages was evaluated using western blot analysis. Prior to their stimulation with LPS for 22 h, macrophages were pretreated with the MSC-CM for 2 h. We define the MSC-CM derived from the D1 cell culture (a bone marrow-derived MSC cell line) as D1-M (CON)— D1 cell conditioned media (control); the MSC-CM derived from the IL4-stimulated D1 cell culture is defined as D1-M (IL4)— IL4-stimulated D1 cell conditioned media.

**Figure 1:**
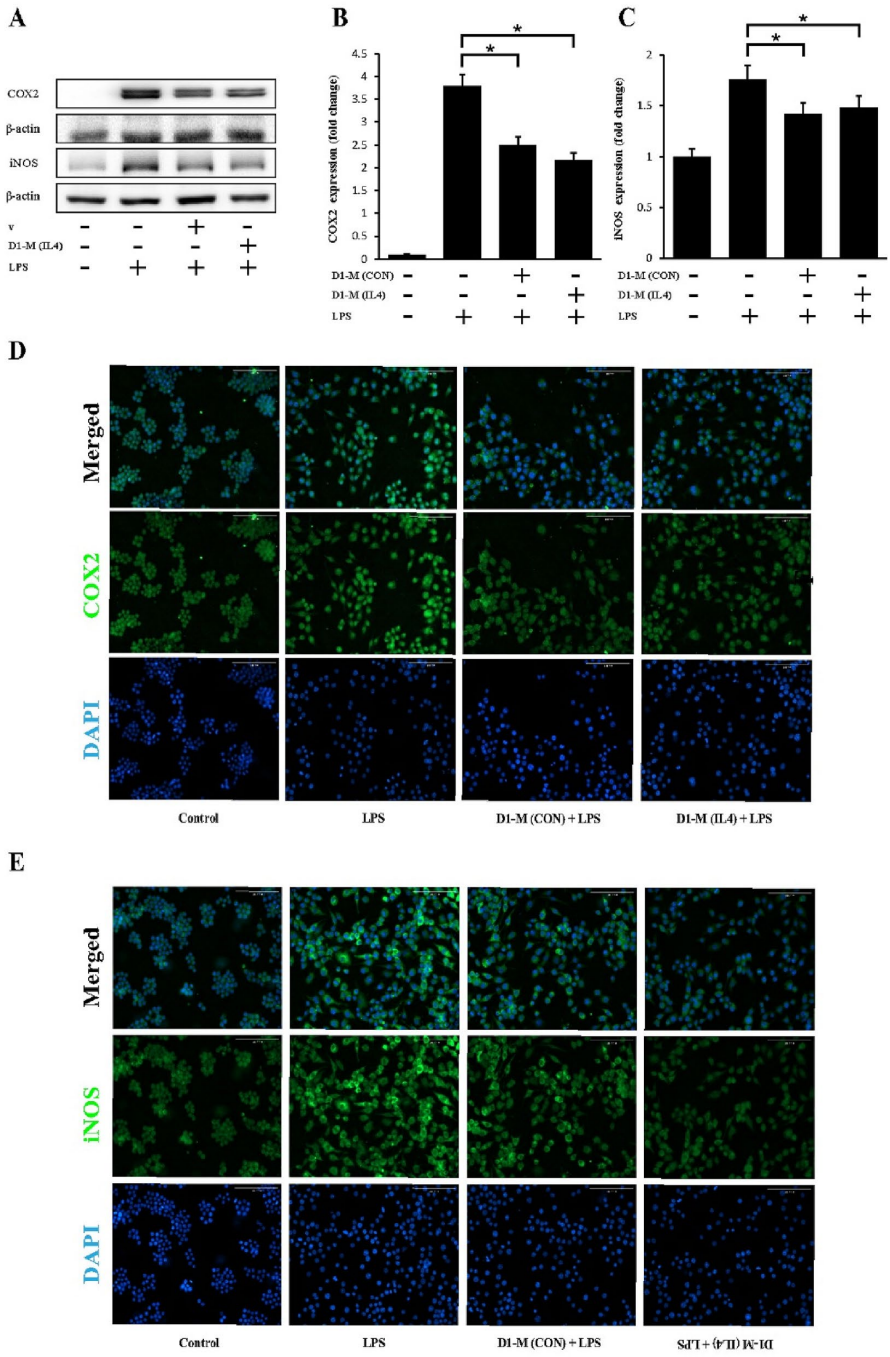
Mesenchymal stem cell conditioned media inhibited the LPS-induced expression of pro-inflammatory enzymes. LPS stimulation effectively upregulated the production of COX-2 and iNOS, which was reduced by pretreatment with MSC-CM (**A**). Representative western blots of COX-2 (**B**) and iNOS (**C**) expression in LPS-stimulated RAW264.7 cells with or without MSC-CM treatment for 24 h; quantitative data representing the average values of three separate experiments. Fluorescence images (× 200) of RAW264.7 cells immunostained for COX-2 (green) and DAPI (blue) (**D**) as well as iNOS (green) and DAPI (blue) (**E**). LPS treatment was performed for 6 h after pretreatment with MSC-CM. The results are representative of three separate experiments (^*^P < 0.05, ^**^P < 0.01 compared with LPS stimulation without MSC-CM). The Fig. 1A and D were cropped to improve the clarity and conciseness of the presentation, and the full-length blots/gels are presented in Supplementary file. MSC-CM, mesenchymal stem cell conditioned media; D1-M (CON), D1 cell media; D1-M (IL4), D1 cell media stimulated with IL4; LPS, lipopolysaccharides; COX-2, cyclooxygenase-2; iNOS, inducible nitric oxide synthase.

LPS stimulation substantially upregulated the expression of COX-2 and iNOS, but pretreatment with MSC-CM decreased LPS-induced COX-2 and iNOS expression (Fig. 1). LPS-stimulated RAW264.7 cells demonstrated an upregulated expression of COX-2 and iNOS, which was inhibited by D1-M (IL4) treatment (Fig. 1A–C).

For the immunofluorescence analysis, macrophages were pretreated with MSC-CM for 24 h and then stimulated with LPS (200 ng/mL) for 6 h. The mean gray values were determined and analyzed. The expression of inflammatory cytokines, such as COX-2 and iNOS, was upregulated in response to LPS stimulation, and was inhibited by pretreatment with MSC-CM (Fig. 1D,E). Further, the COX-2 signal was notably inhibited in the D1-M (IL4) group compared to the D1-M (CON) group. Meanwhile, no significant difference was observed in the iNOS signal between the two groups. These results indicate that treatment with MSC-CM inhibited inflammatory marker expression in the activated macrophages.

### MSC-CM reduced the production of pro-inflammatory cytokines, chemokines, and chemokine receptors

**Figure 2:**
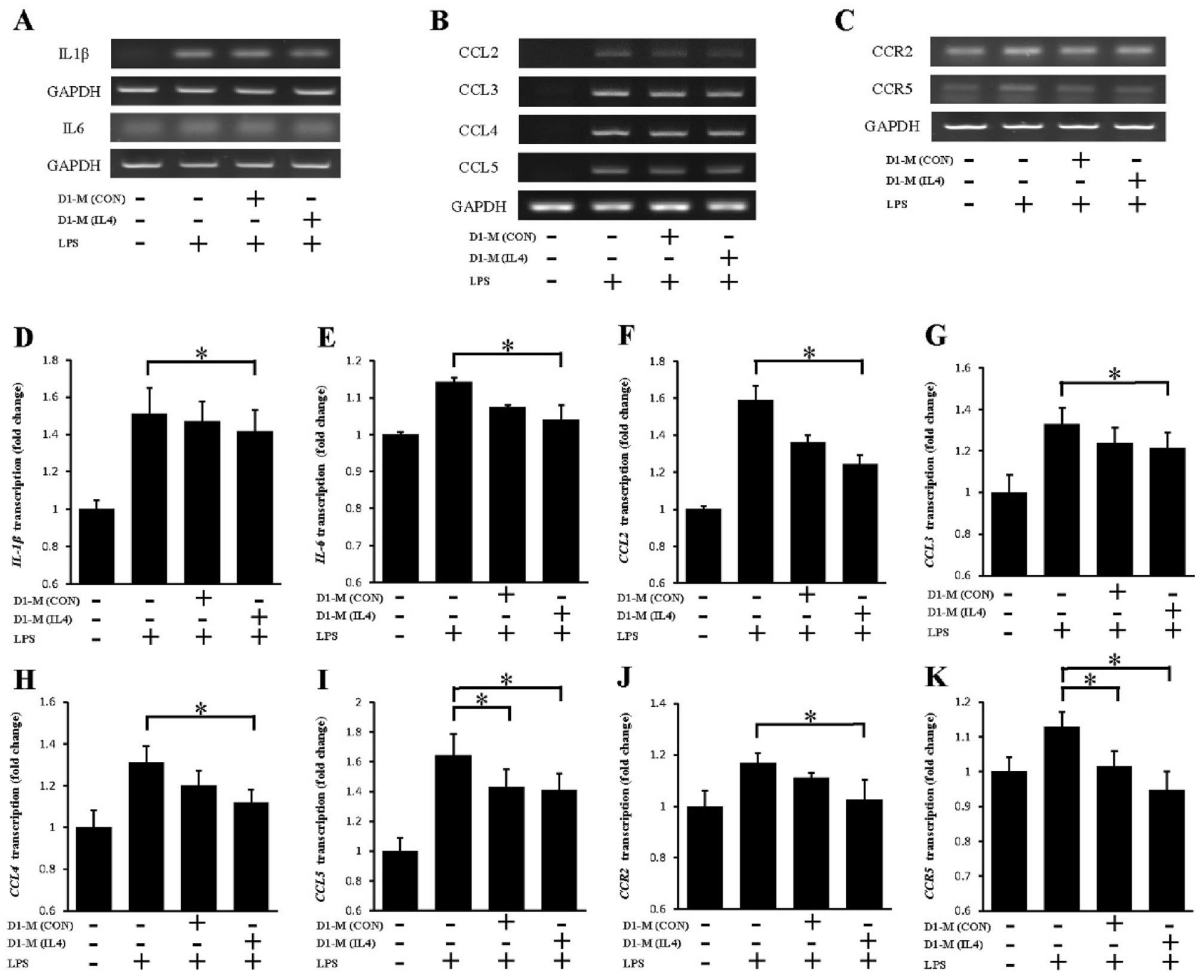
MSC-CM reduced the mRNA transcription of LPS-induced inflammatory cytokines, chemokines, and chemokine receptors in RAW264.7 cells. The expression of inflammatory cytokines and chemokines was analyzed with RT-PCR (**A**–**C**). RAW264.7 cells were pretreated with MSC-CM for 18 h and then stimulated with LPS (200 ng/mL) for 6 h. The mRNA transcription of the inflammatory cytokine genes, *IL1β* (**D**) and *IL6* (**E**), in LPS-stimulated RAW264.7 cells with or without MSC-CM pretreatment. The mRNA transcription of chemokine-encoding genes, such as *CCL2* (**F**), *CCL3* (**G**), *CCL4* (**H**), and *CCL5* (**I**), in LPS-stimulated RAW264.7 cells with or without MSC-CM treatment. The mRNA transcription of the genes of chemokine receptors, such as *CCR2* (**J**) and *CCR5* (**K**), in LPS-stimulated RAW264.7 cells with or without MSC-CM. The data are representative of three separate experiments (^*^P < 0.05 compared to LPS stimulation with or without MSC-CM treatment). The (**A**–**C**) were cropped to improve the clarity and conciseness of the presentation, and the full-length blots/gels are presented in Supplementary file. MSC-CM, mesenchymal stem cell conditioned media; D1-M (CON), D1 cell media; D1-M (IL4), D1 cell media stimulated with IL4; LPS, lipopolysaccharides; IL, interleukin; TNF, tumor necrosis factor; CCL, C–C motif ligand; CCR, C–C motif receptor.

The major pro-inflammatory cytokines (IL1β and IL6), pro-inflammatory chemokines (CCL2, CCL3, CCL4, and CCL5), and chemokine receptors (CCR2 and CCR5) are commonly expressed in LPS-stimulated macrophages. In this study, the effects of MSC-CM on the mRNA transcription of *IL1β*, *IL6*, *CCL2*, *CCL3*, *CCL4*, *CCL5*, *CCR2*, and *CCR5* were examined using RT-PCR. RAW264.7 cells were pretreated with or without MSC-CM for 18 h, and then stimulated by LPS for 6 h. In the LPS-activated RAW264.7 cells, the mRNA transcription of *IL1β, IL6, CCL2, CCL3, CCL4, CCL5, CCR2*, and *CCR5*, was upregulated (Fig. 2A–C). Then the mRNA transcription of *IL1β, IL6, CCL2, CCL3, CCL4, CCL5, CCR2,* and *CCR5* was significantly suppressed following MSC-CM treatment (Fig. 2 A–K). The signal notably decreased in the D1-M (IL4) group compared with that observed in the LPS group with the expression of *IL1β*, *IL6*, *CCL2*, *CCL3*, *CCL4*, and *CCR2*. However, despite this reducing trend, the difference between the LPS and D1-M (CON) groups was not significant (Fig. 2D–G,H,J). For *CCL5* and *CCR5*, no significant differences were observed between the D1-M (IL4) and D1-M (CON) groups, however, both groups showed a significantly reduced expression compared with the LPS group (Fig. 2I, K). These results indicate that the MSC-CM inhibited the mRNA transcription of pro-inflammatory cytokines, chemokines, and chemokine receptors in the LPS-stimulated macrophages.

### MSC-CM inhibited the LPS-induced phosphorylation of MAPKs and NF-κB

**Figure 3:**
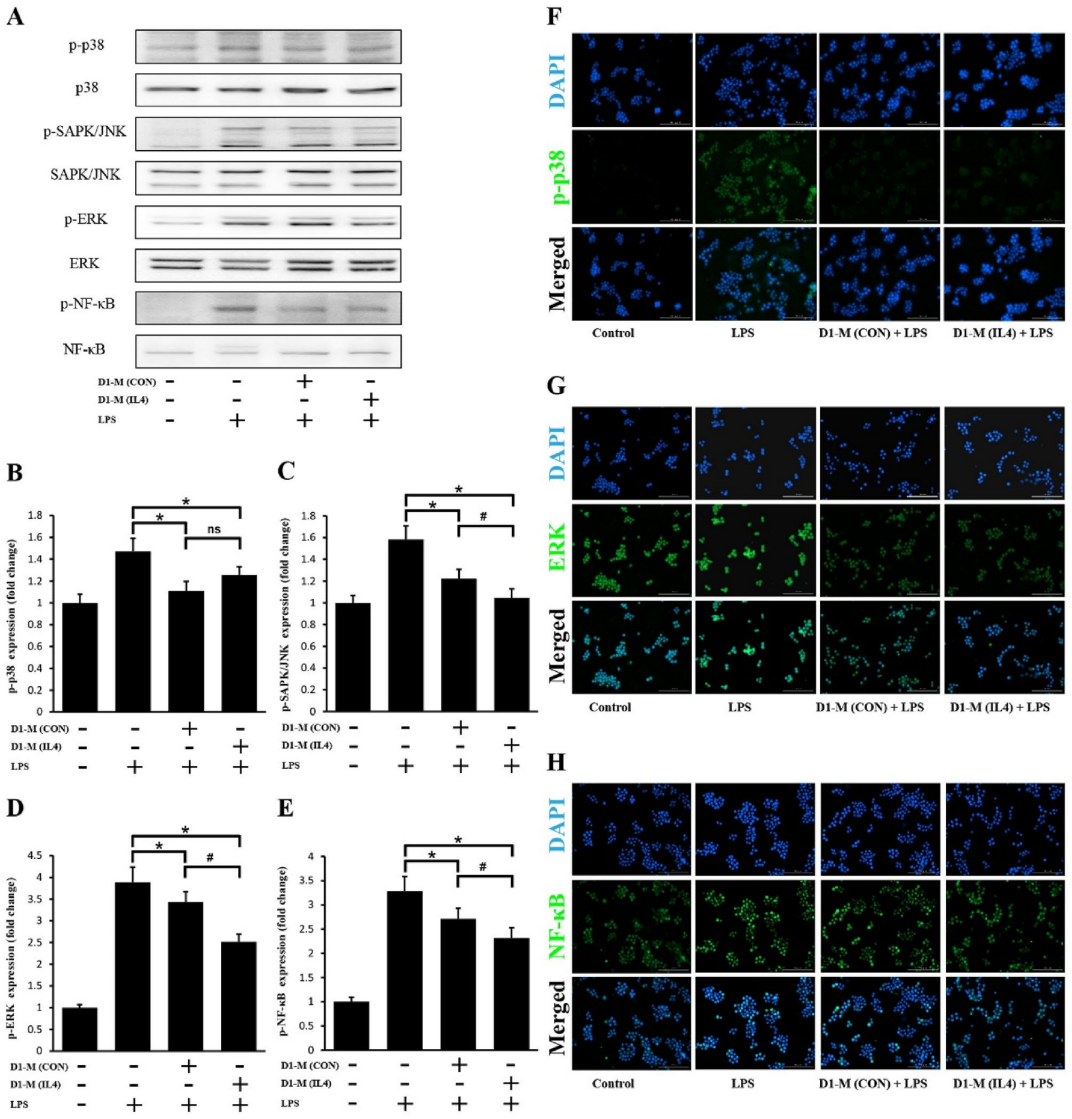
MSC-CM treatment suppressed the LPS-stimulated phosphorylation of p38 MAPK, SAPK/JNK, ERK1/2, and NF-κB (**A**). Representative western blots of anti-p38 and anti-p-p38 (**B**), anti-SAPK/JNK and anti-p-SAPK/JNK (**C**), anti-ERK1/2 and anti-p-ERK1/2 (**D**), and anti-NF-κB and anti-p-NF-κB (**E**) antibody detection in LPS-stimulated RAW264.7 cells with or without MSC-CM treatment for 24 h. For immunofluorescence analysis, RAW264.7 cells were pretreated with MSC-CM for 24 h and then stimulated with LPS (200 ng/mL) for 1 h. Fluorescence images (× 200) of RAW264.7 cells immunostained for p-p38 (green) (**F**), p-ERK1/2 (green) (**G**), and p-NF-κB (green) (**H**) with DAPI counterstain (blue) as control. The MAPK phosphorylation signal was increased by LPS stimulation and suppressed by MSC-CM pretreatment. No such changes were observed for the JNK signal (Supplementary Fig. 2). The results are representative of three separate experiments. The quantitative data represent the average values of three separate experiments (^*^P < 0.05 compared to LPS treatment without MSC-CM; ^#^*P* < 0.05 compared between MSC-CM-treated groups; ns, no statistical difference between MSC-CM-treated groups, *P* > 0.05). The Fig. 3 A was cropped to improve the clarity and conciseness of the presentation, and the full-length blots/gels are presented in Supplementary file. MSC-CM, mesenchymal stem cell-conditioned media; D1-M (CON), D1 cell media; D1-M (IL4), D1 cell media stimulated with IL4; LPS, lipopolysaccharide; SAPK/JNK, stress-activated protein kinase/Jun-amino-terminal kinase; ERK, extracellular signal-regulated kinase; NF-κB, nuclear factor kappa-B.

The anti-inflammatory effects of MSC-CM led to an inhibition of the LPS-stimulated phosphorylation of MAPKs and NF-κB in RAW264.7 cells. Phosphorylation of p38 MAPK, stress-activated　protein　kinase/c-Jun N-terminal　kinase (SAPK/JNK), extracellular regulated protein kinases (ERK) 1/2, and NF-κB was upregulated in RAW264.7 cells after LPS stimulation. Pretreatment with MSC-CM significantly suppressed the LPS-stimulated phosphorylation of p38 MAPK, SAPK/JNK, ERK1/2, and NF-κB (Fig. 3A). The inhibition of SAPK/JNK, ERK1/2, and NF-κB phosphorylation was more pronounced with D1-M (IL4) treatment than D1-M (CON) treatment (Fig. 3C–E). However, lower inhibition of p38 MAPK was observed in the D1-M (IL4) group compared with the D1-M (CON) group; although the difference was not significant (Fig. 3B).

For the immunofluorescence analysis, RAW264.7 cells were pretreated with MSC-CM for 22 h. Subsequently, the cells were stimulated with 200 ng/mL LPS for 1 h. The mean gray values were determined and analyzed. Phosphorylation of p38 MAPK, JNK, ERK1/2, and NF-κB was upregulated after the LPS stimulation of macrophages. Pretreatment with MSC-CM significantly suppressed the LPS-stimulated phosphorylation of p38 MAPK, ERK1/2, and NF-κB (Fig. 3F–H), whereas no significant difference was observed in the p-JNK signal upon treatment with LPS or MSC-CM (Supplementary Fig. 2). Further the D1-M (IL4) and D1-M (CON) groups demonstrated similar signal intensity with respect to p38 MAPK and ERK 1/2. These results indicate that the MAPK and NF-κB signaling pathways may mediate the anti-inflammatory effects of the MSC-CM on the LPS-stimulated RAW264.7 cells.

## Discussion

In this study, the anti-inflammatory effect of D1 cells was studied in LPS-stimulated RAW264.7 macrophage cells by evaluating the gene and protein expression of pro-inflammatory mediators and factors involved in intracellular signaling pathway activation. The MSC-CM did not exert any toxic effects on macrophages, which was confirmed by treatment with different concentrations. PCR and western blot analyses demonstrated that LPS triggered the inflammatory reaction of macrophages, while the D1 cell media suppressed this response. The findings of this study indicate that the anti-inflammatory effects of MSC-CM are mediated via the inhibition of the p38 MAPK, JNK, and ERK signaling pathways and the NF-κB signaling pathway.

Macrophages are important cells that participate in inflammatory diseases^5,30,31^. Macrophages also induce the synovial inflammation and pathological changes of the cartilage and bone characteristic of OA^30^. Tissue-resident macrophages perform major roles in the regulation of tissue inflammation, such as in diabetic glomerular sclerosis^5^. In sepsis-induced lung injury, macrophages defend against invading pathogens during sepsis, but also induce injury to alveolar epithelial cells and capillary endothelial cells^31^. Elucidating the exact mechanism of the recruitment and activation of monocytes and macrophages triggered by damage associated molecular patterns may lead to the development of potential therapies^31–33^.

MSCs possess strong immunomodulatory potential and can regulate adaptive and innate immunity^19,21^. The pro-inflammatory enzymes COX-2 and iNOS cause the upregulation of inflammatory factors in LPS-stimulated macrophages^16^. Gu et al*.* demonstrated that COX-2 levels decreased significantly in cigarette-exposed macrophage cells following treatment with MSCs^34^. Transcription of *iNOS* was reduced in LPS-stimulated MSC-macrophage cocultures^17^. Yang et al*.* demonstrated that iNOS expression in the LPS-stimulated RAW264.7 cells cultured with the primed canine MSC-CM decreased^35^. In the present study, the MSC-CM pretreatment inhibited the expression of the pro-inflammatory enzymes COX-2 and iNOS.

Inflammation is triggered by the activation of pro-inflammatory cytokines IL1β and IL6^20,36,37^ as well as the chemokines CCL2, CCL3, CCL4, and CCL5^38,39^. These cytokines and chemokines are involved in inflammation progression, tissue repair, and immune responses via the recruitment of immune cells, as well as the regulation of autocrine/paracrine activities^39^. Therefore, a novel therapeutic strategy involving the development of an anti-inflammatory agent should target the inhibition of pro-inflammatory cytokines expressed by macrophages^40^. The expression of the pro-inflammatory macrophage marker IL1β is decreased in LPS-stimulated and MSC-treated macrophages compared to the controls^17^. Xu et al*.* reported that IL‐6 expression decreased in macrophages due to LPS-stimulated MSC-derived exosomes^41^. In the current study, the MSC-CM reduced the expression of pro-inflammatory cytokines, such as IL1β and IL6, in LPS-stimulated macrophages. The inhibition effect on LPS-induced IL1β and IL6 production was more pronounced in the D1-M (IL4) group compared with the D1-M (CON) group. The mechanism involving the chemokine alterations, the anti-inflammatory effects, and the activated macrophages has not yet been thoroughly investigated^42^. In addition, various studies have examined the role of chemokines during inflammation in inflammatory diseases^10,43–46^. Chemokine receptors expressed on the surfaces of macrophages induce the accumulation and chemotaxis of chemokines at the inflammation site^47^. Raghu et al*.* demonstrated that CCL2/CCR2 and CCL5/CCR5 were involved in inflammation and tissue damage in human and murine OA via the recruitment of monocytes^11^. Larmonier et al*.* reported that colonic inflammation was inhibited partly in vitro and in vivo through the inhibition of chemokine expression, such as that of CCL3 and CXCL2^45^. Sindhu et al*.* determined that CCL3 and CCL4 contributed to the expression of TNF-α, IL-1β, and IL-6 in activated macrophages and resulted in the development of metabolic inflammation^46^. These findings demonstrate a significant relationship between chemokine concentration and inflammation intensity. In the present study, the MSC-CM suppressed the transcription of *CCL2*, *CCL3*, *CCL4*, *CCL5*, *CCR2*, and *CCR5* in LPS-stimulated macrophages. The D1-M (IL4) group demonstrated a more pronounced inhibition of the LPS-induced transcription of *CCL2*, *CCL3*, *CCL4*, and *CCR2*. No differences were observed in *CCL5* and *CCR5* transcription between the D1-M (IL4) and D1-M (CON) groups. This indicated that MSC-CM treatment inhibited the inflammatory components expressed by LPS-stimulated macrophages by suppressing the mRNA transcription of pro-inflammatory cytokines and chemokines.

**Figure 4:**
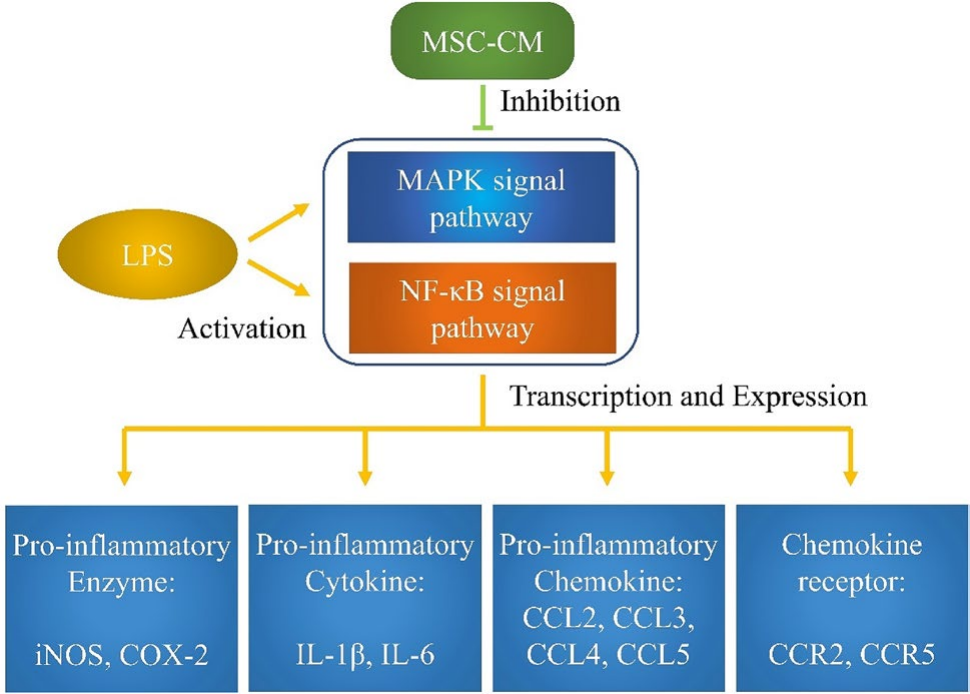
MSC-CM suppressed the gene expression of pro-inflammatory factors via the MAPK and NF-κB signaling pathways to inhibit inflammation. MSC-CM, mesenchymal stem cell conditioned media; MAPK, mitogen-activated protein kinase; SAPK/JNK, stress-activated protein kinase/Jun-amino-terminal kinase; ERK, extracellular signal-regulated kinase; NF-κB, nuclear factor kappa-B; iNOS, inducible nitric oxide; COX-2, cyclooxygenase-2; IL1β, interleukin-1β; CCL, C–C motif ligand; CCR, C–C motif receptor; LPS, lipopolysaccharides.

The MAPK and NF-κB signaling pathways lead to the activation of macrophages^48–51^, which may be inhibited by MSC-CM. A previous study demonstrated that phosphorylation of MAPKs was activated by LPS and was involved in LPS-induced NO production and pro-inflammatory cytokine expression^52^. Zheng et al*.* determined that in LPS-stimulated RAW264.7 cells, an MSC-conditioned medium might reduce NF-κB signaling pathway activation^49^. The MAPK and NF-κB signaling pathways play important roles in mediating the anti-inflammatory effects of MSCs on activated macrophages^53–55^. The results obtained in the present study were consistent with those of previous studies. JNK expression remained unchanged in all groups across several replicate experiments. This might be attributed to the use of an incompatible antibody for immunofluorescence analysis. These results demonstrated that LPS treatment stimulated the phosphorylation of p38 MAPK, JNK, and ERK1/2 as well as that of NF-κB, which is consistent with the results of previous studies. Thus, our findings demonstrate that the MSC-CM exerts anti-inflammatory effects by mediating the MAPK and NF-κB signaling pathways (Fig. 4). In addition, the MSC-CM with IL4 stimulation showed more potent anti-inflammatory effects than that without IL4 stimulation.

This study had some limitations. First, the exact components primarily affecting the macrophages were not identified. However, as reported by previous studies, MSCs could express the anti-inflammatory cytokines IL4, IL10, and IL13 that might suppress macrophage activation^15–17^. Second, several methods for studying the effects of MSCs on macrophages have been reported in literature, including co-culture techniques with or without Transwell, utilization of MSC-CM, and that of the MSC-CM-derived exosomes^17,41,49,56^. In this study, we only used the MSC-CM method; thus, additional methods should be used in further research to confirm the anti-inflammatory effects of MSCs. Third, the stimulation of MSCs by the cytokines IL10, IL13, TNF-α, and IL1β should be considered in order to determine whether these factors also enhance the anti-inflammatory effects of MSCs along with IL4. Finally, we did not include a comparison group of macrophages treated directly with IL4. Some IL4 could have remained despite considering the possibility that IL4 in MSC-CM may have affected macrophages and changed the media before MSC-CM collection.

## Conclusion

The MSC-CM suppressed the production of pro-inflammatory compounds in the LPS-stimulated macrophages. The inhibition of pro-inflammatory cytokine and chemokine production was associated with the inhibition of the MAPK and NF-κB pathways in LPS-stimulated macrophages. The IL4-treated MSCs demonstrated a more potent anti-inflammatory effect on LPS-induced macrophage activation. These findings may have implications for potential therapeutic strategies for inflammatory disease.

## Materials and methods

### Cell culture and treatment

**Figure 5:**
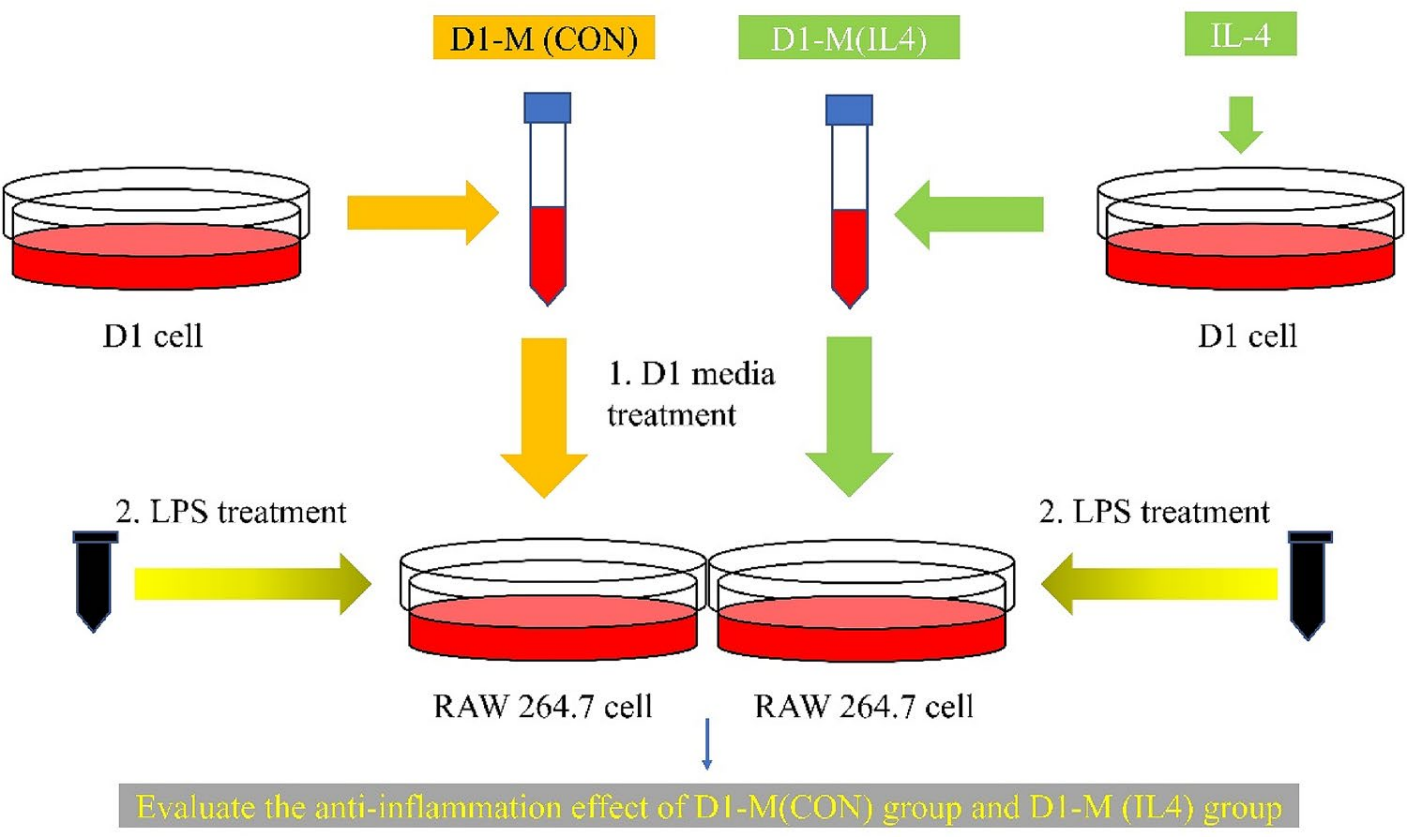
Procedure for the preparation of D1 cell media (D1-M) and the treatment of RAW264.7 cells. The D1 cells were stimulated with or without IL4 and incubated for 24 h, followed by media change and additional incubation for 24 h. After obtaining D1-M, RAW264.7 culture medium was replaced with a mixture of D1-M and complete media prepared at certain ratios (1:100, 1:10, 1:1, and 1) after incubation for 24 h, following the seeding of RAW264.7 cells. After media replacement, RAW264.7 cells were incubated with D1-M for 24 h, and LPS (working concentration: 200 ng/mL) was used to treat the RAW264.7 cells for the last 1, 6, and 22 h of incubation for different assays. D1-M (CON) represents D1 cell medium without IL4 treatment; D1-M (IL4) represents D1 cell medium stimulated with IL4.

RAW264.7 cells are murine macrophages that were obtained from the American Type Culture Collection (ATCC, Manassas, VA, USA). In this study, RAW264.7 cells were cultured with 5% CO_2_ at 37 °C in DMEM supplemented with 10% FBS, 200 μg/mL streptomycin, 200 IU/mL penicillin, 4 mM L-glutamine, and 1 mM sodium pyruvate (complete medium). LPS was diluted to obtain the final concentration of 200 ng/mL in the medium (Fig. 5).

A bone marrow-derived MSC cell line, D1 ORL UVA (D1) (ATCC #CRL-12424), was purchased from the ATCC. Primary mouse D1 cells were purchased from the ATCC and incubated with or without 20 ng/mL IL4 for stimulation for 24 h, then incubated for an additional 24 h following media change. The D1 cell supernatant was collected and classified into two groups; D1-M (IL4) and D1-M (CON) (Fig. 5).

### Cytotoxicity assay

Cytotoxicity was evaluated via colorimetric MTT assay. RAW264.7 cells were incubated in a 96-well cell culture plate and 100 µl complete medium overnight. Different ratios of D1-M and DMEM media (1:100 to 1) were added to each well, and the plate was then incubated for 24 h at 37 °C in an incubator. After D1-M treatment, the supernatant was discarded, and MTT solution (0.5 mg/mL) was pipetted into each well. Following incubation for 2 h at 37 ℃, the MTT solution was carefully discarded, and the formazan deposit was dissolved with dimethyl sulfoxide. A purple-colored solution was obtained. Optical density (OD) of the formazan solution was estimated by measuring the absorbance at 570 nm using the Synergy™ HTX Multi-Mode Microplate Reader.

### Reverse transcription-PCR

RAW264.7 were pretreated by a 1:1 ratio of D1-M and media for 18 h. The cells were then incubated with or without LPS for 6 h. After 24 h of incubation, the supernatant was discarded via centrifugation, and a total RNA extraction reagent (RNAiso Plus. Takara, Japan) was used to isolate total RNA from the cells according to the manufacturer’s instructions. Subsequently, 1 μg of isolated total RNA was carefully reverse transcribed with oligo dT (Bioneer, Daejeon, Korea), *Taq* DNA polymerase, dNTP, and reaction buffer (AccuPower® PCR PreMix kit, Bioneer, Daejeon, Korea) to synthesize cDNA. To evaluate mRNA transcription regarding target inflammatory cytokines and chemokines, the primer sequences specific for mouse target genes were designed (Supplement Table 1). The MyGenie 96 gradient thermal cycler system, AccuPower® RT PreMix kit (Bioneer, Daejeon, Korea), and the primers were used to amplify the cDNA. The PCR products were visualized using NEOgreen DNA staining reagent (NEO Science, Daejeon, Korea) and a NaBI Gel-doc system.

### Western blot analysis

Before LPS (200 ng/mL) stimulation, cells were pretreated with or without a 1:1 ratio of D1-M and media. Following incubation with or without pre-treatment, a total incubation for 24 h at 37 °C was performed. The LPS stimulation were accomplished at the last 1 h (for MAPKs and NF-κB) and 22 h (for COX-2 and iNOS) of incubation. Subsequently, the cells were rinsed twice with cold phosphate-buffered saline (PBS). The total cells were lysed with radio-immunoprecipitation assay (RIPA) buffer (Sigma, St. Louis, MO, USA) and treated with protease inhibitor cocktail (GenDEPOT, Katy, TX, USA) at 4 °C in an icebox for 30 min. The lysates were concentrated through the centrifuge, which was conducted at 12,000 × *g* and 4 °C for 15 min. After centrifugation, using the BCA assay kit (Thermofisher, Rockford, IL, USA) to measure the protein concentration of cell lysates according to the manufacturer’s instructions. The protein was divided using 10% or 15% sodium dodecyl SDS-PAGE with equivalent amounts of protein samples, and a polyvinylidene difluoride (PVDF) membrane was used to transfer proteins. The 5% skim milk solution was used to block membranes for 1 h at room temperature and the blot was cut prior to hybridization with antibodies during blotting. Subsequently, with the following primary antibodies: anti-COX-2 (Abcam, Cambridge, UK), anti-iNOS (Abcam, Cambridge, UK), anti-β-actin (Santa Cruz Biotechnology, Dallas, TX, USA), anti-p38, anti-p-p38, anti-SAPK/JNK, anti-p-SAPK/JNK, anti-ERK1/2, and anti-p-ERK1/2 (from Cell Signaling Technology, Danvers, MA, USA), PVDF membranes were incubated overnight at 4 °C (Supplement Table 2). Secondary antibodies used the horseradish peroxidase (HRP) conjugated anti-rabbit, anti-mouse, and anti-American hamster antibodies (Santa Cruz Biotechnology). Bands were visualized with the Cooled CCD Gel Imaging System. Pre-stained protein markers (HiQ™ Blueye Prestained Protein Marker, Bio-D) were used for the determination of molecular weight.

### Immunofluorescence

Cells were seeded on coverslips in 12 well plates, and were incubated in the absence or presence of a 1:1 ratio of D1-M and DMEM media for 24 h. This was followed by LPS (200 ng/mL) treatment during the last 1 h (for MAPKs and NF-κB) or 6 h (for COX-2 and iNOS) of incubation at 37 °C. After fixation with paraformaldehyde (4%) and permeabilization with Triton X-100 along with washing with 1 × PBS, cells were blocked with the 1% BSA for 1 h at 37 °C incubation and stained overnight with primary antibodies at 4 °C (Supplement Table 3). The cells were then stained with Alexa 488 dye-labeled secondary antibodies (Cell Signaling Technology, Danvers, MA, USA) (Supplement Table 3). All primary antibodies were used at a ratio of 1:400. All secondary antibodies were used at a ratio of 1:2000 for cultured RAW264.7. Nuclei of the cells were stained with 4′,6 -diamidino-2-phenylindole (DAPI)-containing mounting solution (VECTASHIELD® Antifade Mounting Medium with DAPI, Vector, Burlingame, CA, USA), and the images were obtained using an augmented microscopy system with a microplate reader and imager software. For semi-quantitative analysis of the fluorescence, the mean gray values were detected and compared between each group with Image J (Collins, 2007). Under 200 × magnification imaging, three fields of each cell slide were selected, and the positively stained macrophages were calculated to obtain a mean value (Supplement Table 4). Sections were randomly coded and scored by a blinded observer with three sections per group.

### Statistical analysis

The data obtained in this study were assessed via SPSS, and were analyzed using a one-way analysis of variance (ANOVA) and post-hoc Tukey’s test. The data were expressed as mean ± standard deviation (mean ± SD). The alpha level for all tests was 0.05, with two-tailed, and statistically significant was considered as *p* < 0.05.

## Supplementary Information

Supplementary Information 1.

Supplementary Information 2.

Supplementary Information 3.
