# Supplementary material for: Anti-inflammatory effects of mesenchymal stem cell-conditioned media inhibited macrophages activation in vitro
Source: Sci Rep. 2022 Mar 19;12:4754. doi: 10.1038/s41598-022-08398-4 (PMC8934344; doi:10.1038/s41598-022-08398-4)
Supplement: Supplementary file 1 — Supplementary Information 1. [file 41598_2022_8398_MOESM1_ESM.docx]

**Supplementary Figures**

**
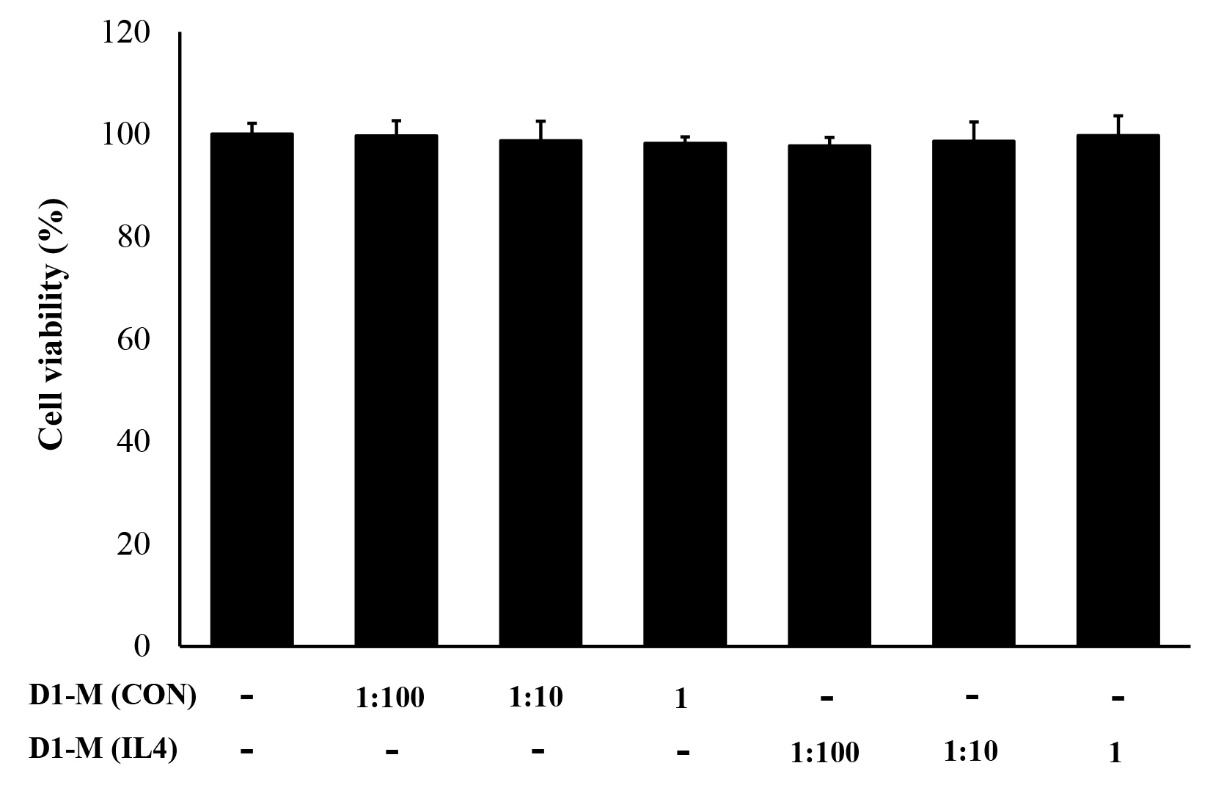
**

**Figure S1.** MSC-CMs did not exert any cytotoxic effects on RAW264.7 cells. RAW264.7 cells were incubated into 96-well plates. Different ratios of MSC-CM and complete media (1:100, 1:10, and 1) were added, and viable cell numbers were evaluated using MTT assay after 24 h incubation. Results are represented as viable cell numbers described in percentages in comparison to the control group. It represents an average of four replicate data from three separate experiments. MSC-CM, Mesenchymal stromal cell conditioned media; D1-M (CON), D1 cell media; D1-M (IL4), D1 cell media stimulated with IL-4.


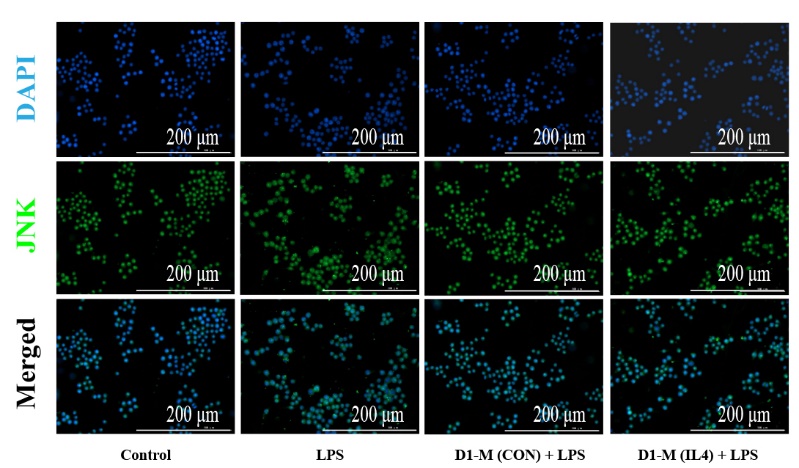


**Figure S2.** MSC-CM didn't inhibit LPS-stimulated phosphorylation of JNK in RAW264.7 cells in immunofluorescence results. RAW264.7 cells were pre-treated with MSC-CM for 24 h, and then stimulated by LPS (200 ng/mL) for 1 h. (A-C) Fluorescence images (x200) of RAW264.7 immunostained for p-JNK (green) with DAPI counterstain (blue) of control. No changes were observed for the JNK signal. MSC-CM, Mesenchymal stromal cell conditioned media; D1-M (CON), D1 cell media; D1-M (IL4), D1 cell media stimulated with IL-4; LPS, Lipopolysaccharides; JNK, Jun-amino-terminal kinase.
