# Supplementary material for: Anti-inflammatory effects of mesenchymal stem cell-conditioned media inhibited macrophages activation in vitro
Source: Sci Rep. 2022 Mar 19;12:4754. doi: 10.1038/s41598-022-08398-4 (PMC8934344; doi:10.1038/s41598-022-08398-4)
Supplement: Supplementary file 2 — Supplementary Information 2. [file 41598_2022_8398_MOESM2_ESM.docx]

Supplementary Information

**Table of Contents**

| 1. RT-PCR results in text ......................................................................................... | S2-S6 |
| --- | --- |
| 2. Western blot results in text ................................................................................... | S7-S18 |

**1. RT-PCR results in text:**


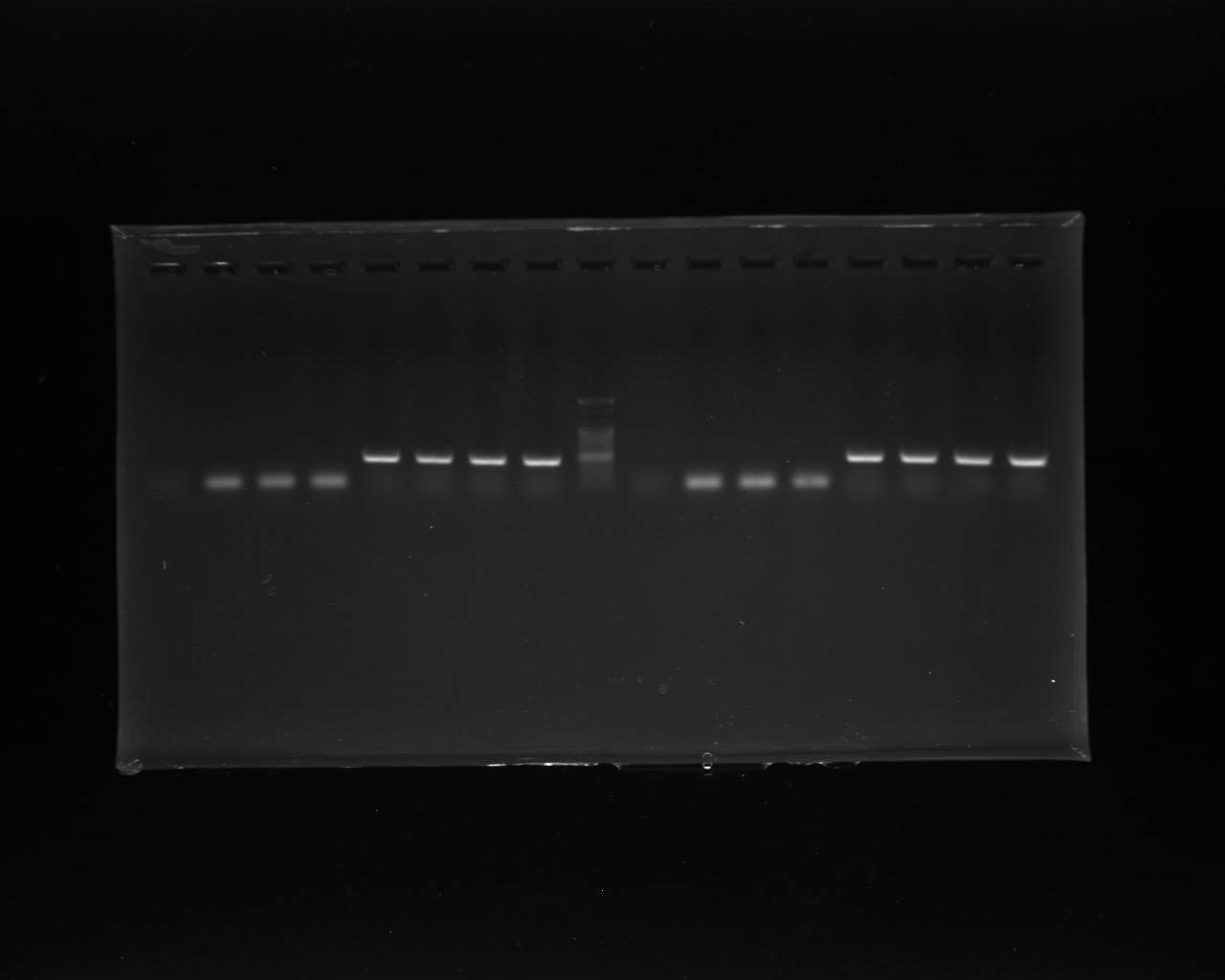


Control

LPS

D1-M (CON) + LPS

D1-M (IL4) + LPS

IL-1β

GAPDH

Supplementary file 1: The presentation of the RT-PCR result refers to *IL-1β*. Each dataset contained 4 group: “control”, “LPS”, “D1-M (CON) +LPS”, and “D1-M (IL4) +LPS” groups, from left side to right side. The PCR products were visualized using NEOgreen DNA staining reagent (NEO Science, Daejeon, Korea) and a NaBI Gel-doc system (NEO Science, Daejeon, Korea).


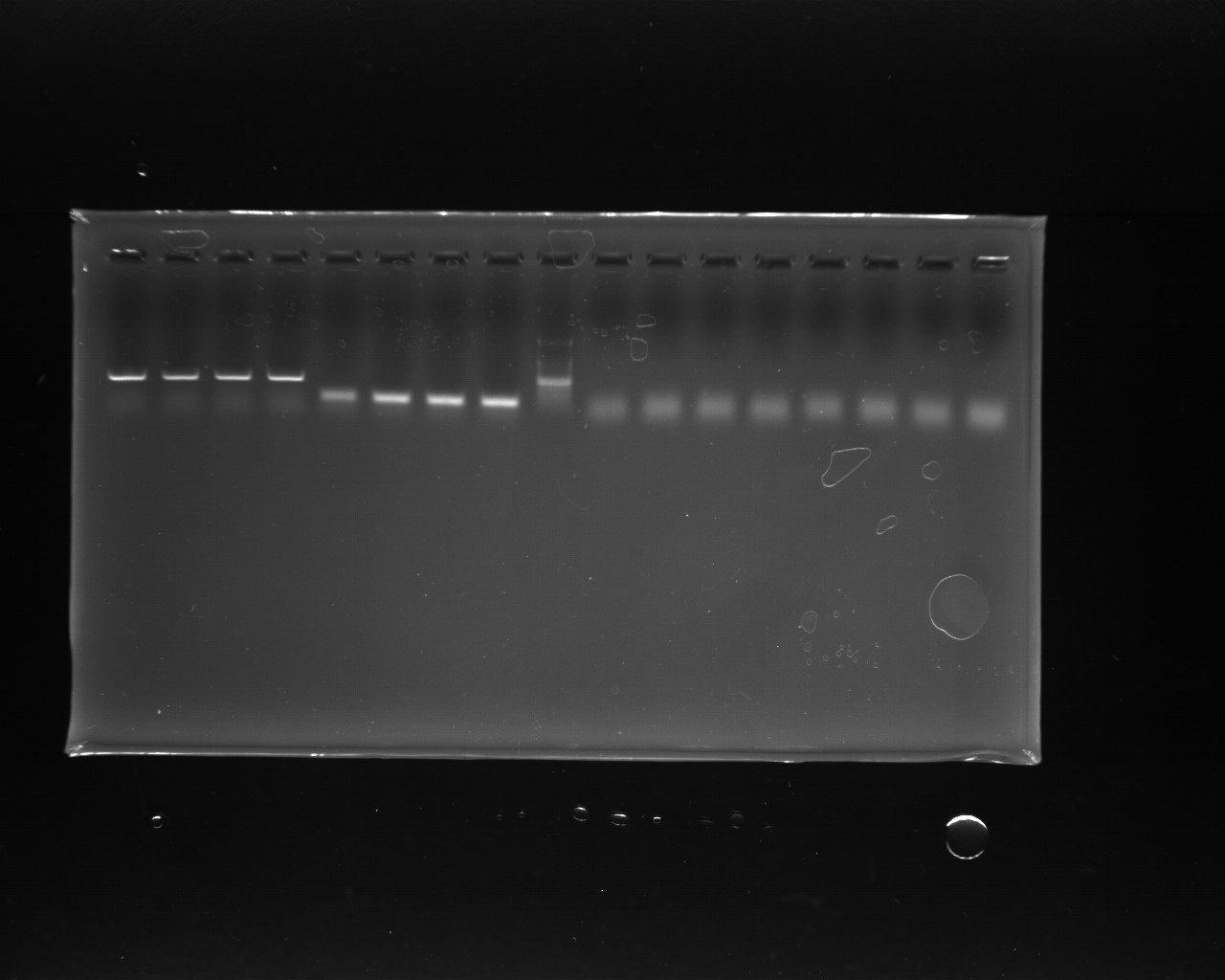


IL-6

Control

LPS

D1-M (CON) + LPS

D1-M (IL4) + LPS

GAPDH

Supplementary file 2: The presentation of the RT-PCR result refers to *IL-6*. Each dataset contained 4 group: “control”, “LPS”, “D1-M (CON) +LPS”, and “D1-M (IL4) +LPS” groups, from left side to right side. The PCR products were visualized using NEOgreen DNA staining reagent (NEO Science, Daejeon, Korea) and a NaBI Gel-doc system (NEO Science, Daejeon, Korea).


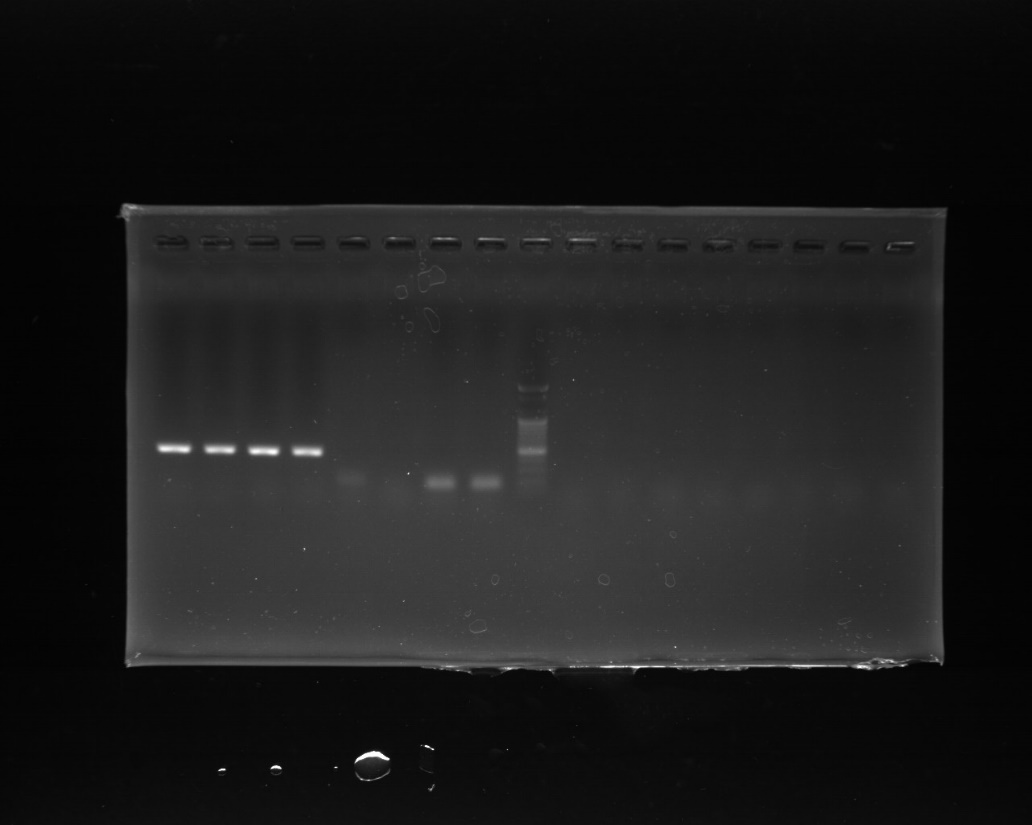


Control

LPS

D1-M (CON) + LPS

D1-M (IL4) + LPS

GAPDH


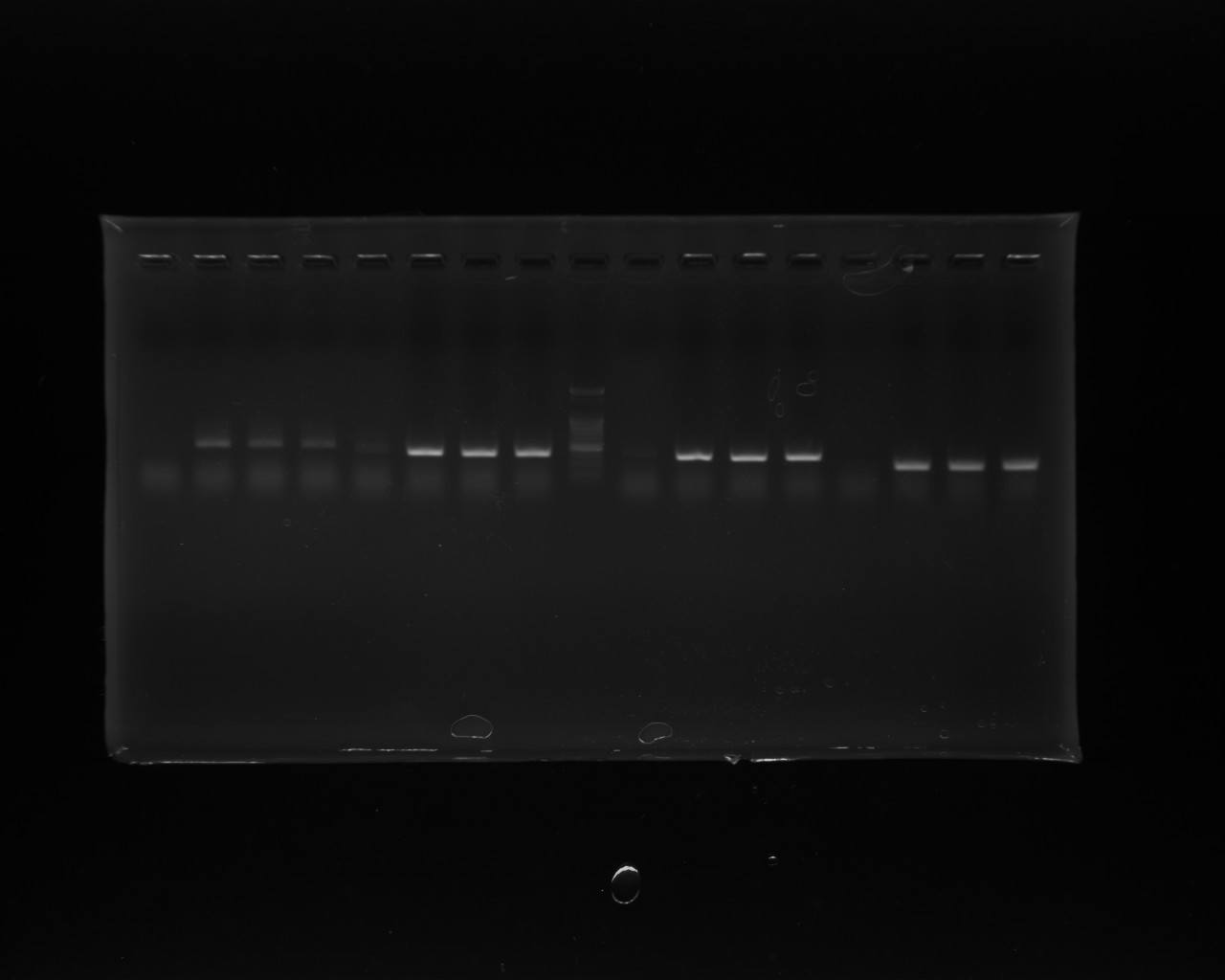


Control

LPS

D1-M (CON) + LPS

D1-M (IL4) + LPS

CCL2

CCL3

CCL4

CCL5

Supplementary file 3: The presentation of the RT-PCR result refers to *CCL2*, *CCL3*, *CCL4*, and *CCL5*. Each dataset contained 4 group: “control”, “LPS”, “D1-M (CON) +LPS”, and “D1-M (IL4) +LPS” groups, from left side to right side. The samples derive from the same experiment and those gels were processed electrophoresis in parallel. The PCR products were visualized using NEOgreen DNA staining reagent (NEO Science, Daejeon, Korea) and a NaBI Gel-doc system (NEO Science, Daejeon, Korea).


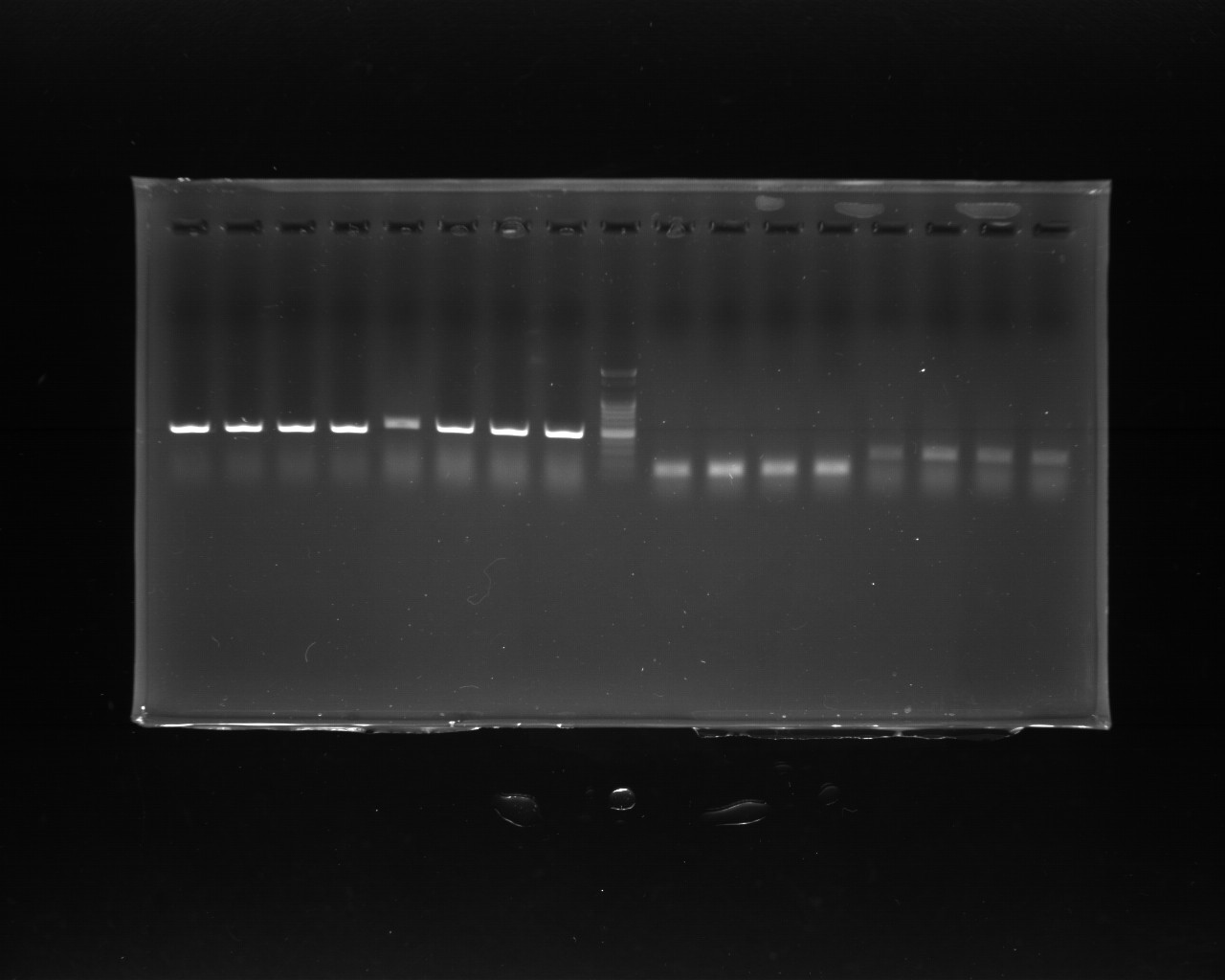


Control

LPS

D1-M (CON) + LPS

D1-M (IL4) + LPS

CCR5

CCR2

GAPDH

Supplementary file 4: The presentation of the RT-PCR result refers to *CCR2* and *CCR5*. Each dataset contained 4 group: “control”, “LPS”, “D1-M (CON) +LPS”, and “D1-M (IL4) +LPS” groups, from left side to right side. The PCR products were visualized using NEOgreen DNA staining reagent (NEO Science, Daejeon, Korea) and a NaBI Gel-doc system (NEO Science, Daejeon, Korea).

**2. Western blot results in text:**


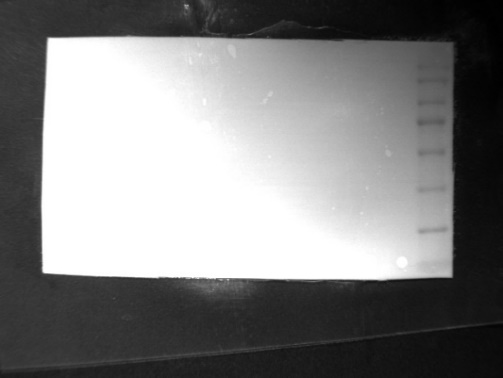

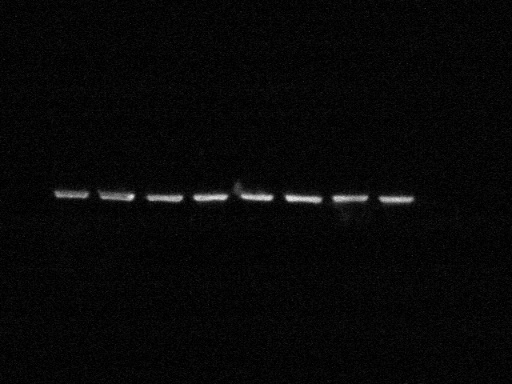


**A**

**B**

**A**


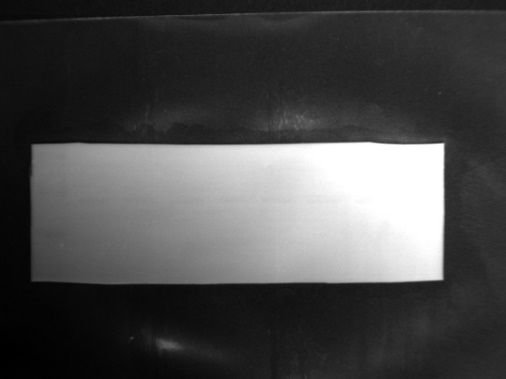

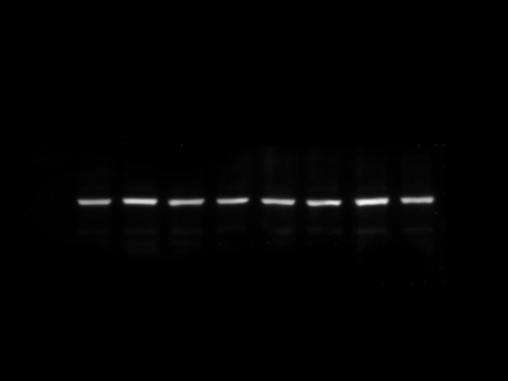


**D**

**C**

**A**


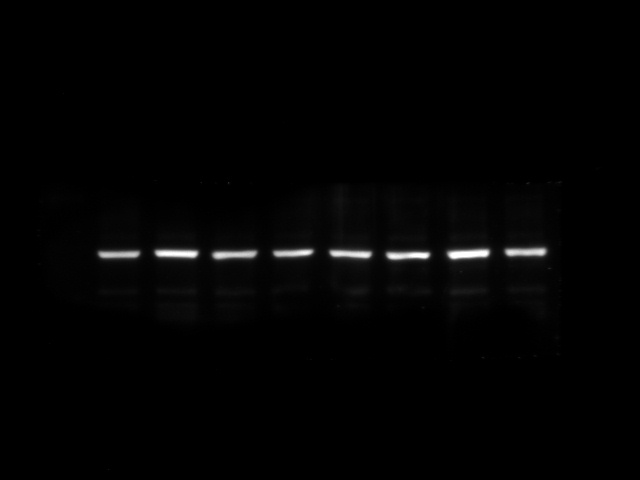


**E**

43 kDa

Control

LPS

D1-M (CON) + LPS

D1-M (IL4) + LPS

β-actin

Supplementary file 5: The presentation of the Western blot result refers to β-actin (iNOS) (43 kDa). The blot was cut prior to hybridisation with antibodies during blotting. Each dataset contained 4 group: “control”, “LPS”, “D1-M (CON) +LPS”, and “D1-M (IL4) +LPS” groups, from left side to right side. Bands were visualized with the Cooled CCD Gel Imaging System (AE-9100N Ez-Capture, ATTO, Tokyo). A: Full-length membrane, B: Exposure time:4s, C: Cut membrane:30s, D: Exposure time:45s.


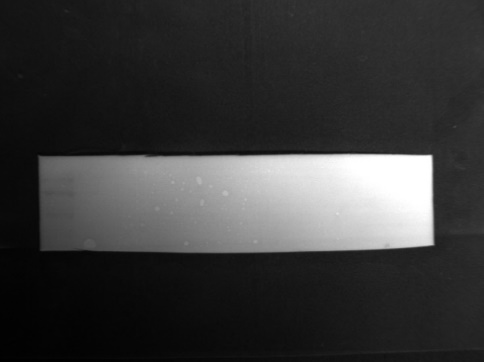

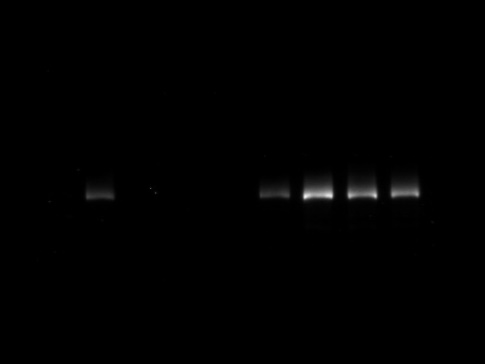


**A**

**B**


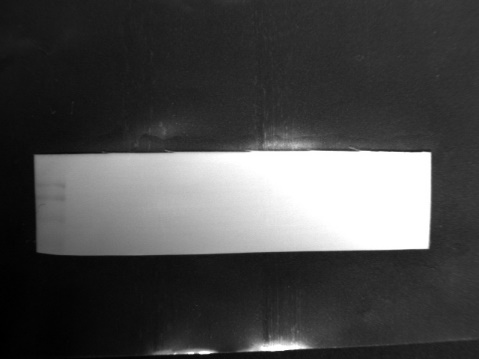

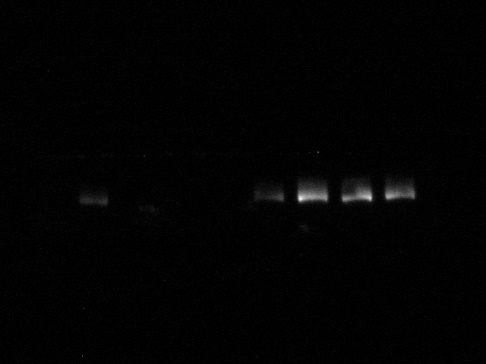


**D**

**C**


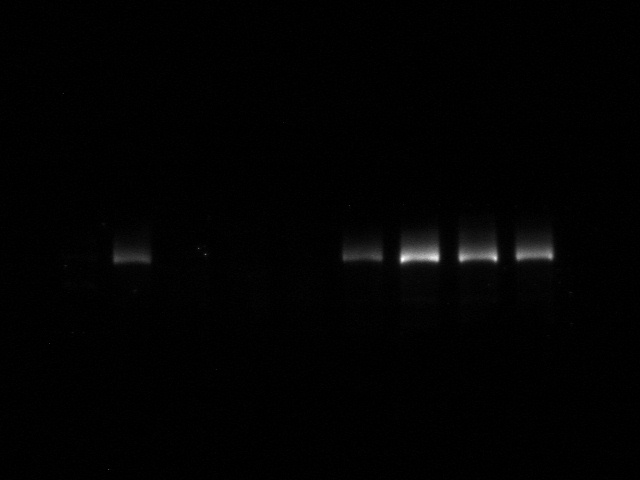


**E**

Control

LPS

D1-M (CON) + LPS

D1-M (IL4) + LPS

131 kDa

iNOS

Supplementary file 6: The presentation of the Western blot result refers to iNOS (131 kDa). Each dataset contained 4 group: “control”, “LPS”, “D1-M (CON) +LPS”, and “D1-M (IL4) +LPS” groups, from left side to right side. Bands were visualized with the Cooled CCD Gel Imaging System (AE-9100N Ez-Capture, ATTO, Tokyo). A: Membrane of 1^st^ exposure, B: Exposure time of 1^st^ try:2min, A: Membrane of 2^nd^ exposure, B: Exposure time of 2^nd^ try:3min, C: Exposure time of 1^st^ try:30s.


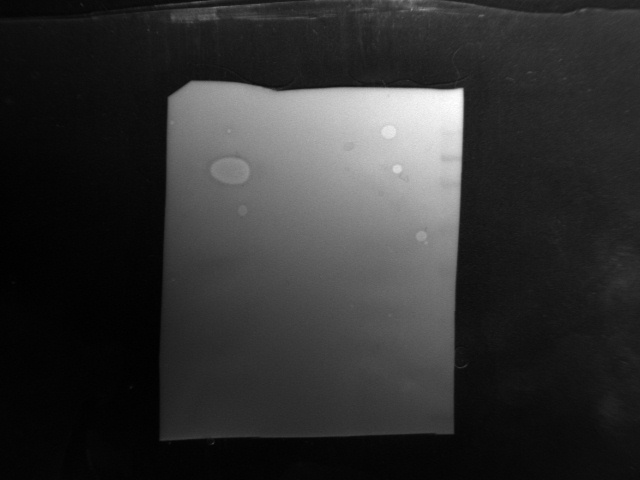


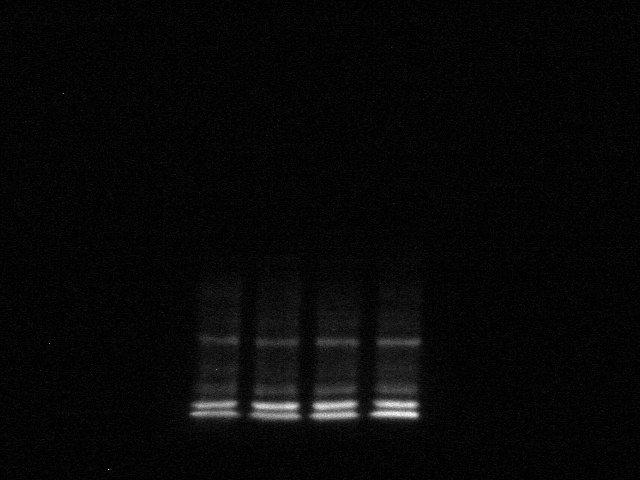


Control

LPS

D1-M (CON) + LPS

D1-M (IL4) + LPS

43 kDa

β-actin

Supplementary file 7: The presentation of the Western blot result refers to β-actin (COX-2) (43 kDa). Each dataset contained 4 group: “control”, “LPS”, “D1-M (CON) +LPS”, and “D1-M (IL4) +LPS” groups, from left side to right side. Bands were visualized with the Cooled CCD Gel Imaging System (AE-9100N Ez-Capture, ATTO, Tokyo).


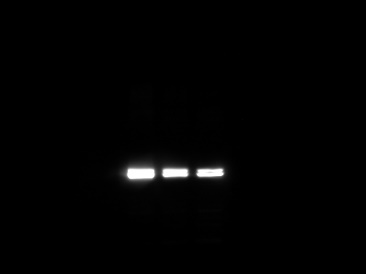

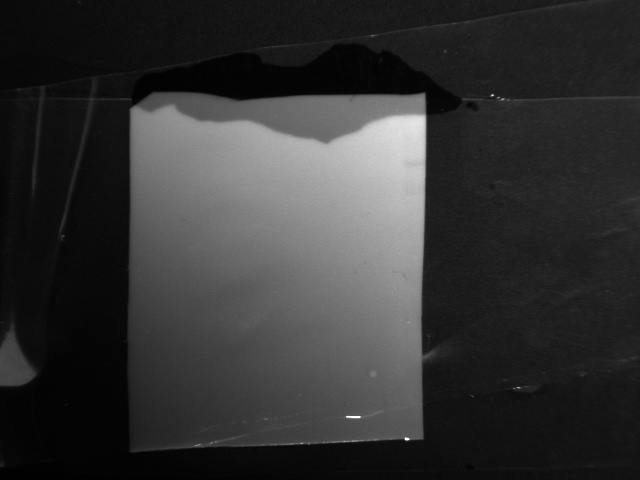

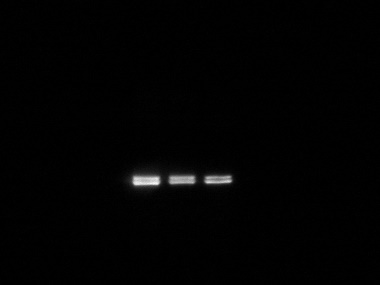


**C**

**BB**

**A**


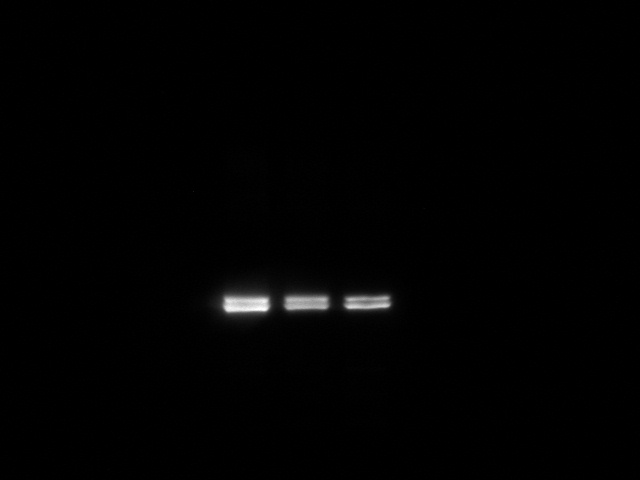


**D**

Control

LPS

D1-M (CON) + LPS

D1-M (IL4) + LPS

69 kDa

COX-2

Supplementary file 8: The presentation of the Western blot result refers to COX-2 (69 kDa). Each dataset contained 4 group: “control”, “LPS”, “D1-M (CON) +LPS”, and “D1-M (IL4) +LPS” groups, from left side to right side. Bands were visualized with the Cooled CCD Gel Imaging System (AE-9100N Ez-Capture, ATTO, Tokyo). A: Membrane, B: Exposure time:1min, C: Exposure time:3s, D: Exposure time:10s.


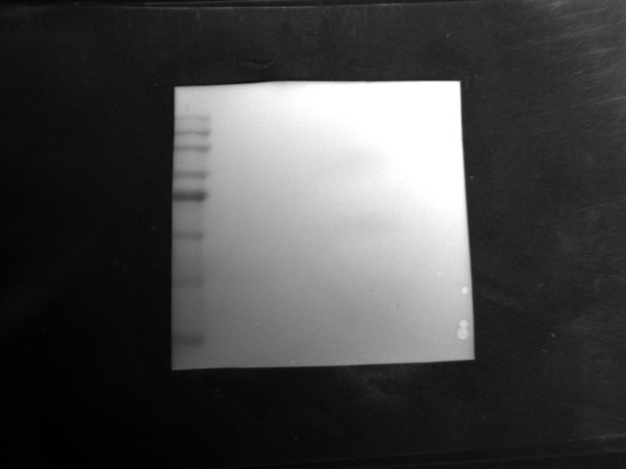

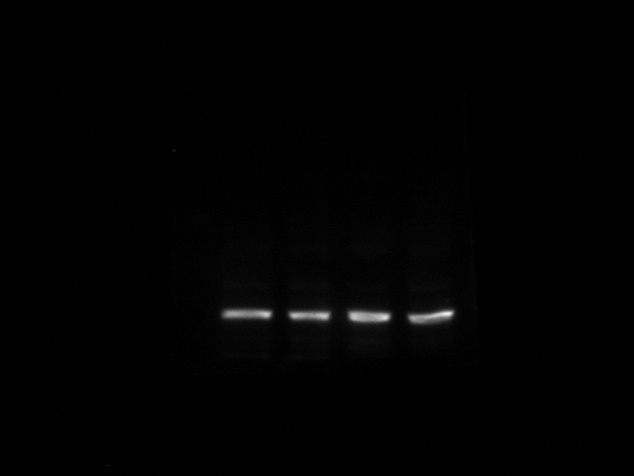


**B**

**A**


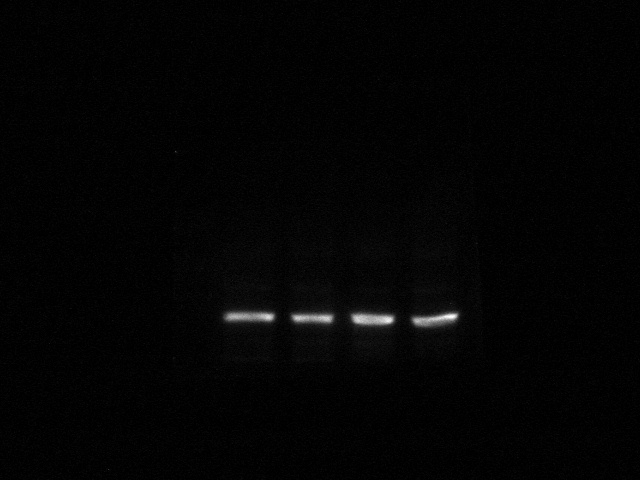


**C**

p38

Control

LPS

D1-M (CON) + LPS

D1-M (IL4) + LPS

40 kDa

Supplementary file 9: The presentation of the Western blot result refers to p38 (40 kDa). Each dataset contained 4 group: “control”, “LPS”, “D1-M (CON) +LPS”, and “D1-M (IL4) +LPS” groups, from left side to right side. Bands were visualized with the Cooled CCD Gel Imaging System (AE-9100N Ez-Capture, ATTO, Tokyo). A: Membrane, B: Exposure time:30s, C: Exposure time:10s.


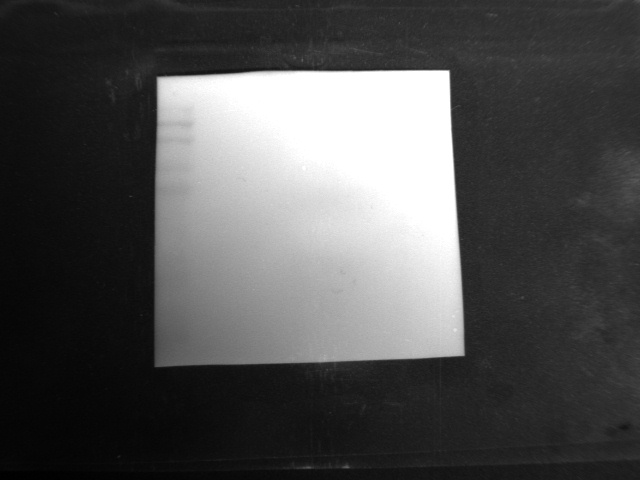


Control

LPS

D1-M (CON) + LPS

D1-M (IL4) + LPS

43 kDa


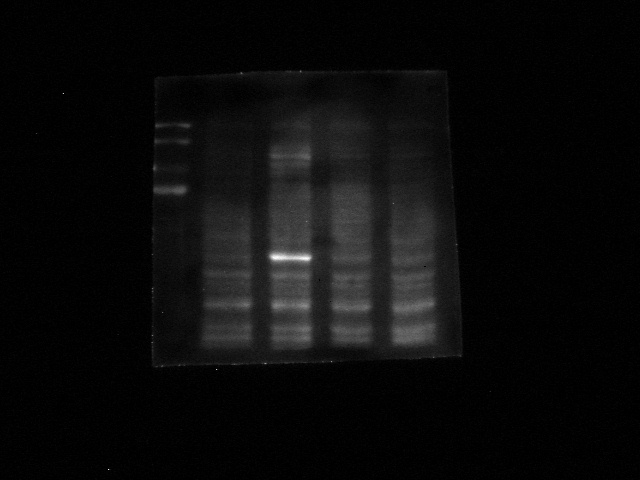


p-p38

Supplementary file 10: The presentation of the Western blot result refers to p-p38 (43 kDa). Each dataset contained 4 group: “control”, “LPS”, “D1-M (CON) +LPS”, and “D1-M (IL4) +LPS” groups, from left side to right side. Bands were visualized with the Cooled CCD Gel Imaging System (AE-9100N Ez-Capture, ATTO, Tokyo).


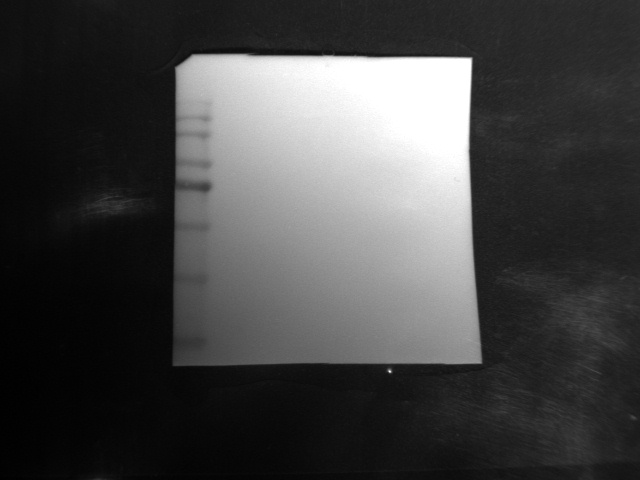


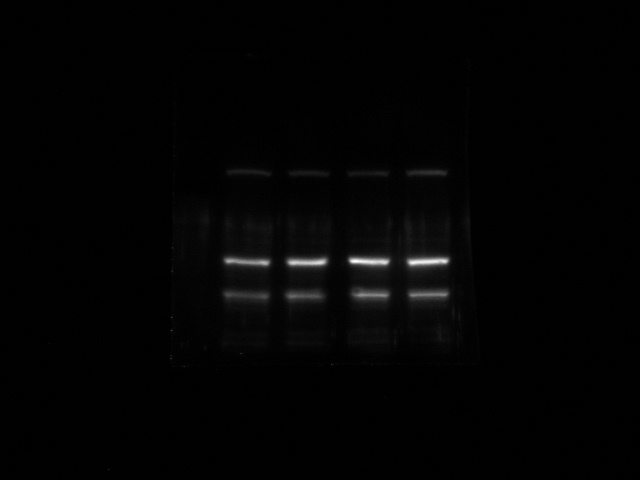


SAPK/JNK

Control

LPS

D1-M (CON) + LPS

D1-M (IL4) + LPS

54 kDa

46 kDa

Supplementary file 11: The presentation of the Western blot result refers to SAPK/JNK (46 kDa, 54 kDa). Each dataset contained 4 group: “control”, “LPS”, “D1-M (CON) +LPS”, and “D1-M (IL4) +LPS” groups, from left side to right side. Bands were visualized with the Cooled CCD Gel Imaging System (AE-9100N Ez-Capture, ATTO, Tokyo).


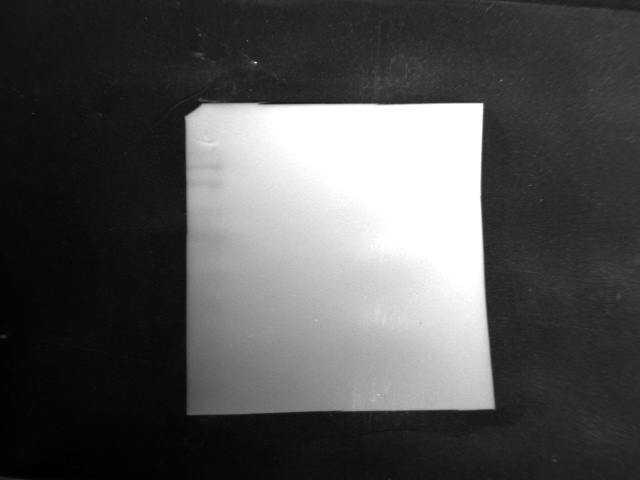


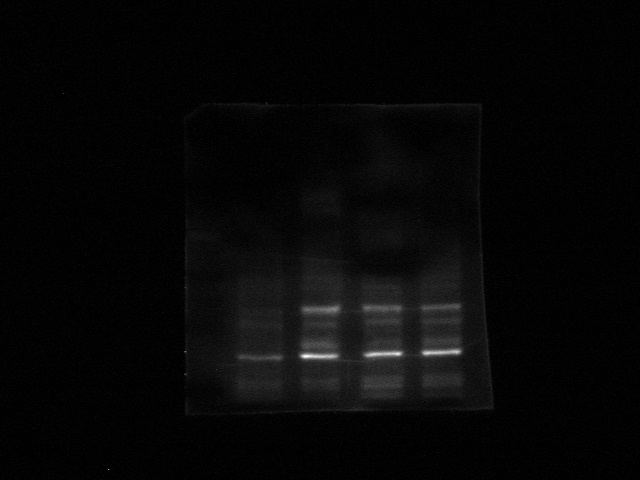


Control

LPS

D1-M (CON) + LPS

D1-M (IL4) + LPS

54 kDa

46 kDa

p-SAPK/JNK

Supplementary file 12: The presentation of the Western blot result refers to p-SAPK/JNK (46 kDa, 54 kDa). Each dataset contained 4 group: “control”, “LPS”, “D1-M (CON) +LPS”, and “D1-M (IL4) +LPS” groups, from left side to right side. Bands were visualized with the Cooled CCD Gel Imaging System (AE-9100N Ez-Capture, ATTO, Tokyo).


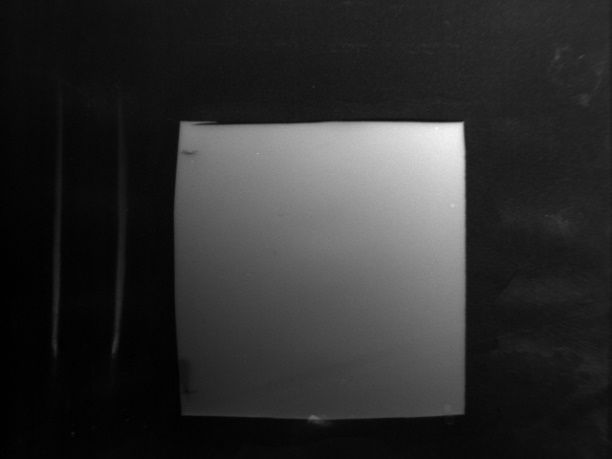

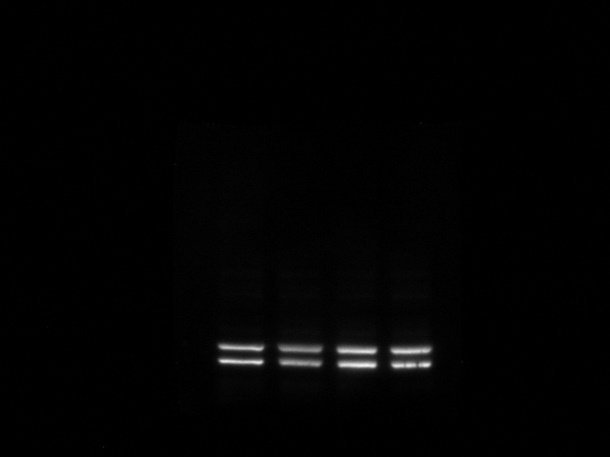


**B**

**A**


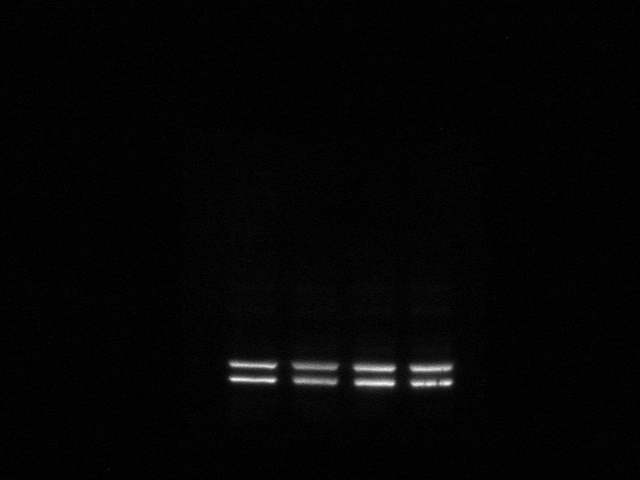


**C**

Control

LPS

D1-M (CON) + LPS

D1-M (IL4) + LPS

44 kDa

42 kDa

ERK

Supplementary file 13: The presentation of the Western blot result refers to ERK (42 kDa, 44 kDa). Each dataset contained 4 group: “control”, “LPS”, “D1-M (CON) +LPS”, and “D1-M (IL4) +LPS” groups, from left side to right side. Bands were visualized with the Cooled CCD Gel Imaging System (AE-9100N Ez-Capture, ATTO, Tokyo). A: Membrane, B: Exposure time: 30s, C: Exposure time: 10s.


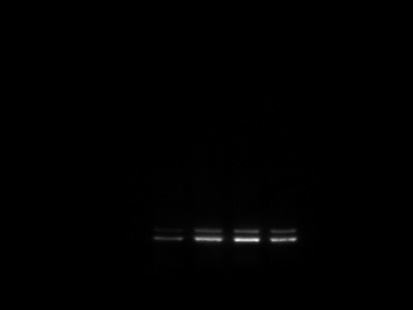

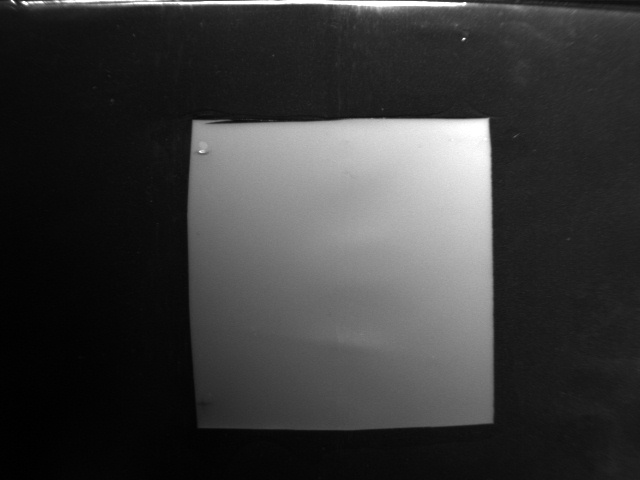

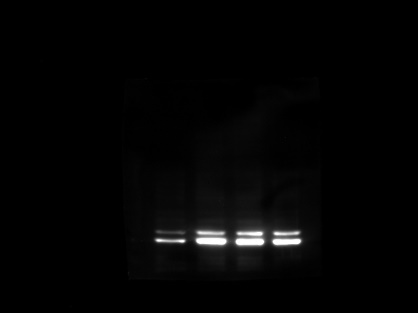


**C**

**B**

**A**


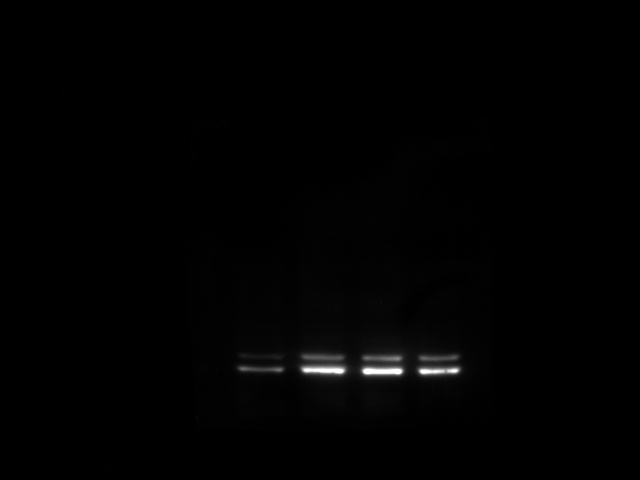


**D**

Control

LPS

D1-M (CON) + LPS

D1-M (IL4) + LPS

44 kDa

42 kDa

p-ERK

Supplementary file 14: The presentation of the Western blot result refers to p-ERK (42 kDa, 44 kDa). Each dataset contained 4 group: “control”, “LPS”, “D1-M (CON) +LPS”, and “D1-M (IL4) +LPS” groups, from left side to right side. Bands were visualized with the Cooled CCD Gel Imaging System (AE-9100N Ez-Capture, ATTO, Tokyo). A: Membrane, B: Exposure time:10s, C: Exposure time:3 mins, D: Exposure time:1 min.


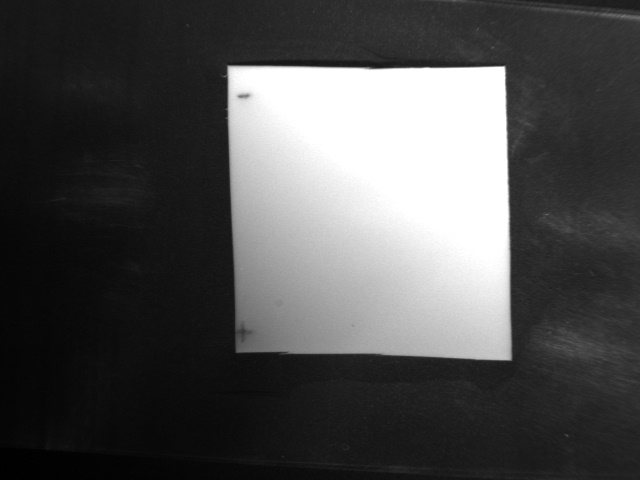


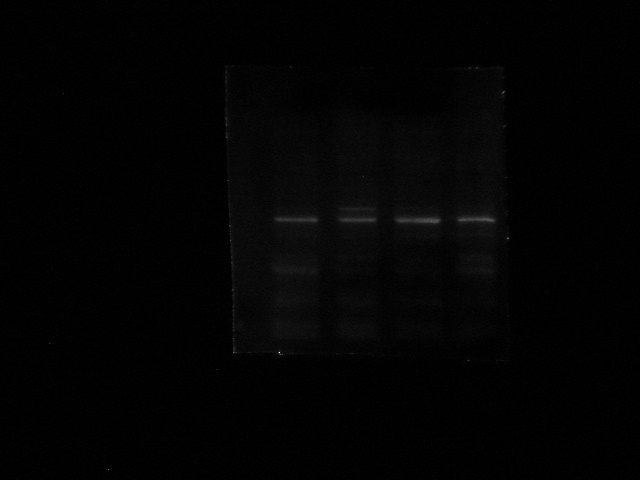


Control

LPS

D1-M (CON) + LPS

D1-M (IL4) + LPS

75 kDa

NF-κB

Supplementary file 15: The presentation of the Western blot result refers to NF-κB (predicted size: 65 kDa, observed size: 75 kDa). Each dataset contained 4 group: “control”, “LPS”, “D1-M (CON) +LPS”, and “D1-M (IL4) +LPS” groups, from left side to right side. Bands were visualized with the Cooled CCD Gel Imaging System (AE-9100N Ez-Capture, ATTO, Tokyo).


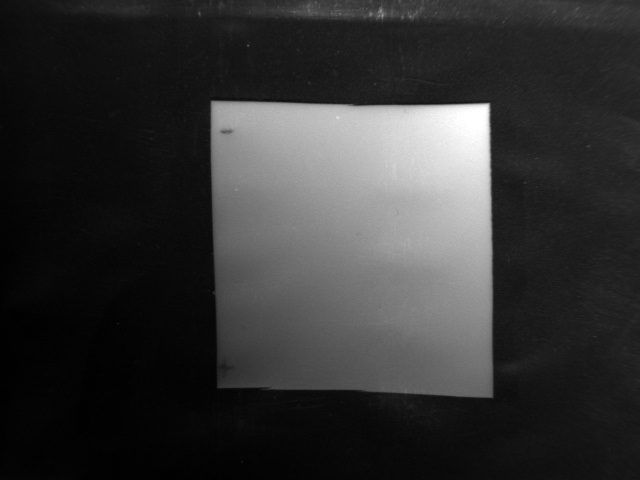


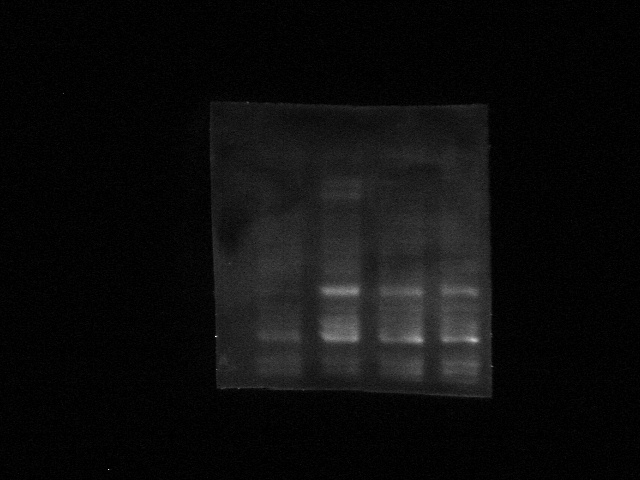


Control

LPS

D1-M (CON) + LPS

D1-M (IL4) + LPS

65 kDa

p-NF-κB

Supplementary file 16: The presentation of the Western blot result refers to p- NF-κB (65kDa). Each dataset contained 4 group: “control”, “LPS”, “D1-M (CON) +LPS”, and “D1-M (IL4) +LPS” groups, from left side to right side. Bands were visualized with the Cooled CCD Gel Imaging System (AE-9100N Ez-Capture, ATTO, Tokyo).
