# Supplementary material for: Anti-inflammatory effects of mesenchymal stem cell-conditioned media inhibited macrophages activation in vitro
Source: Sci Rep. 2022 Mar 19;12:4754. doi: 10.1038/s41598-022-08398-4 (PMC8934344; doi:10.1038/s41598-022-08398-4)
Supplement: Supplementary file 3 — Supplementary Information 3. [file 41598_2022_8398_MOESM3_ESM.docx]

**Table S1. Primers used in RT-PCR**

| **Target** | **Primer sequence (5'- 3')** | **Tm (**℃**)** | **Cycle** |
| --- | --- | --- | --- |
| IL-1β | F： CTACCTGTGTCTTTCCCGTGG | 52.5 | 25 |
| (664bp) | R： CCAGCAGGTTATCATCATC |  |  |
| IL-6 | F： CCTTCCTACCCCAATTTCCA | 52.5 | 25 |
| (465bp) | R： CGCACTAGGTTTGCCCACTA |  |  |
| CCL2 | F： CACTCACCTGCTGCTACTCATTCAC | 58.8 | 30 |
| (813bp) | R： GGATTCACAGAGAGGGAAAAATGG |  |  |
| CCL3 | F： CGGAAGATTCCACGCCAATTC | 58.5 | 30 |
| (657bp) | R： GGTTGAGGAACGTGTCCTGAAG |  |  |
| CCL4 | F： CCCACTTCCTGCTGTTTCTCTTAC | 57.5 | 30 |
| (839bp) | R： AGCAGAGAAACAGCAATGGTGG |  |  |
| CCL5 | F： CCACGTCAAGGAGTATTTCTACACC | 57.5 | 30 |
| (373bp) | R： CTGGTTTCTTGGGTTTGCTGTG |  |  |
| CCR2 | F： GTTACCTCAGTTCATCCA | 49.5 | 30 |
| (117bp) | R： CAAGGCTCACCATCATCGTAGTC |  |  |
| CCR5 | F： CACTGCTGCCTAAACCCTGT | 53.8 | 30 |
| (249bp) | R： TTCCTACTCCCAAGCTGCAT |  |  |
| GAPDH | F： ATCACTGCCACCCAGAAGAC | 58 | 28 |
| (827bp) | R： ATGAGGTCCACCACCCTGTT |  |  |

Abbreviation: IL-1β, Interleukin-1β; CCL, C-C motif ligand; CCR, C-C motif receptor; GAPDH, Glyceraldehyde 3-phosphate dehydrogenase.

**Table S2. Antibodies used in Western blot**

| **1'st antibody** | **Host** | **Conjugation** |
| --- | --- | --- |
| Anti-iNOS | Rabbit |  |
| Anti-COX2 | Rabbit |  |
| Anti-β-actin | Mouse |  |
| Anti-phospho-p38 | Rabbit |  |
| Anti-p38 | Rabbit |  |
| Anti-phospho-JNK1/2 | Rabbit |  |
| Anti-JNK1/2 | Rabbit |  |
| Anti-phospho-ERK1/2 | Rabbit |  |
| Anti-ERK1/2 | Rabbit |  |
| Anti-phospho-NF-κB | Rabbit |  |
| Anti-NF-κB | Rabbit |  |
| Anti-Mouse | Recombinant protein | HRP |
| Anti-Rabbit | Mouse | HRP |

Abbreviation: iNOS, Inducible nitric oxide; COX2, Cyclooxygenase-2; IL, Interleukin; JNK, Jun-amino-terminal kinase; ERK, Extracellular signal-regulated kinase; NF-κB, Nuclear factor kappa-B; HRP, Horseradish peroxidase.

**Table S3. Antibodies used in Immunofluorescence**

| **Antibody** | **Conjugation** | **Host** |
| --- | --- | --- |
| Anti-iNOS | - | Mouse |
| Anti-COX2 | - | Rabbit |
| Anti-phospho-p38 | - | Rabbit |
| Anti-phospho-SAPK/JNK | - | Rabbit |
| Anti-phospho-ERK1/2 | - | Rabbit |
| Anti-phospho-NF-κB | - | Rabbit |
| Anti-Rabbit | Alexa-488 | - |
| Anti-Mouse | Alexa-488 | - |

Abbreviation: iNOS, Inducible nitric oxide; COX2, Cyclooxygenase-2; SAPK/JNK, Stress-activated protein kinase/Jun-amino-terminal kinase; ERK, Extracellular signal-regulated kinase; NF-κB, Nuclear factor kappa-B.

**Table S4. Mean gray values were compared in Immunofluorescence**

| **group** | **Control** | **LPS** | **D1-M (CON)** | **D1-M (IL4)** | **p value^*^** | **p value^&^** | **p value^#^** |
| --- | --- | --- | --- | --- | --- | --- | --- |
| iNOS | 53.2 ± 2.5 | 85.3 ± 5.4 | 59.7 ± 3.3 | 51.1 ± 3.2 | <0.05 | <0.05 | <0.05 |
| COX2 | 63.5 ± 3.7 | 97.2 ± 6.5 | 53.5 ± 4.5 | 51.6 ± 5.3 | <0.05 | <0.05 | ns |
| Phospho-p38 | 59.1 ± 3.2 | 101.8 ± 6.3 | 56.2 ± 7.5 | 54.4 ± 7.9 | <0.05 | <0.05 | ns |
| Phospho-SAPK/JNK | 56.7 ± 4.5 | 95.3 ± 7.7 | 53.7 ± 6.5 | 52.1 ± 8.2 | <0.05 | <0.05 | ns |
| Phospho-ERK1/2 | 81.8 ± 7.7 | 80.4 ± 7.9 | 87.3 ± 6.8 | 88.8 ± 6.3 | ns | ns | ns |
| Phospho-NF-κB | 55.5 ± 6.6 | 90.3 ± 7.2 | 57.6 ± 9.1 | 55.7 ± 5.2 | <0.05 | <0.05 | ns |

p value^*^: LPS treatment without MSC-CM compared to LPS treatment with D1-M (CON)

p value^&^: LPS treatment without MSC-CM compared to LPS treatment with D1-M (IL4)

p value^#^: Compared between MSC-CM treated groups

Abbreviation: iNOS, Inducible nitric oxide; COX2, Cyclooxygenase-2; SAPK/JNK, Stress-activated protein kinase/Jun-amino-terminal kinase; ERK, Extracellular signal-regulated kinase; NF-κB, Nuclear factor kappa-B; MSC-CM, Mesenchymal stromal cell conditioned media; D1-M (CON), D1 cell media; D1-M (IL4), D1 cell media stimulate with IL-4; LPS, Lipopolysaccharides; ns: no statistical difference.
